# Sequence Analysis of the Segmental Duplication Responsible for Paris *Sex-Ratio* Drive in *Drosophila simulans*

**DOI:** 10.1534/g3.111.000315

**Published:** 2011-10-01

**Authors:** Lucie Fouvry, David Ogereau, Anne Berger, Frederick Gavory, Catherine Montchamp-Moreau

**Affiliations:** * Laboratoire Evolution, Génomes et Spéciation, CNRS, 91198 Gif-sur-Yvette, France; † Université Paris-Sud, 91405 Orsay, France; ‡ CEA-GENOSCOPE - Centre National de Séquençage, 91057 Evry Cedex, France

**Keywords:** segmental duplication, meiotic drive, *sex-ratio*, *D. simulans*

## Abstract

*Sex-ratio* distorters are X-linked selfish genetic elements that facilitate their own transmission by subverting Mendelian segregation at the expense of the Y chromosome. Naturally occurring cases of sex-linked distorters have been reported in a variety of organisms, including several species of *Drosophila*; they trigger genetic conflict over the sex ratio, which is an important evolutionary force. However, with a few exceptions, the causal loci are unknown. Here, we molecularly characterize the segmental duplication involved in the Paris *sex-ratio* system that is still evolving in natural populations of *Drosophila simulans*. This 37.5 kb tandem duplication spans six genes, from the second intron of the *Trf2* gene (TATA box binding protein-related factor 2) to the first intron of the *org-1* gene (optomotor-blind-related-gene-1). Sequence analysis showed that the duplication arose through the production of an exact copy on the template chromosome itself. We estimated this event to be less than 500 years old. We also detected specific signatures of the duplication mechanism; these support the Duplication-Dependent Strand Annealing model. The region at the junction between the two duplicated segments contains several copies of an active transposable element, *Hosim1*, alternating with 687 bp repeats that are noncoding but transcribed. The almost-complete sequence identity between copies made it impossible to complete the sequencing and assembly of this region. These results form the basis for the functional dissection of Paris *sex-ratio* drive and will be valuable for future studies designed to better understand the dynamics and the evolutionary significance of sex chromosome drive.

Meiotic drive is a phenomenon by which one member of a pair of alleles or chromosomes of a heterozygous individual is preferentially transmitted to the next generation—a phenomenon that is in violation of Mendel’s first law (Sandler and Novitski 1957). Many examples of meiotic drive have been reported in fungi, plants, insects, worms, and mammals (Atlan *et al.* 1997; Lyttle 1991); *sex-ratio* drive specifically refers to meiotic drive in which the cheater allele is sex linked and is expressed only in heterogametic individuals, resulting in a skewed offspring sex ratio.

X chromosome drive was first observed in males of *Drosophila obscura* (Gershenson 1928) and has since been documented in a number of dipteran species, mainly within the *Drosophila* genus (Jaenike 2001). In *Drosophila*, the *sex-ratio* phenotype is usually associated with X chromosome rearrangements. Inversions of varying complexity, which presumably keep the elements contributing to the drive together, impede genetic dissection in most of the species (De Carvalho and Klaczko 1992; Dyer *et al.* 2007; Hauschteck-Jungen 1990; Prakash 1974; Stalker 1961; Voelker 1972). High-resolution genetic mapping has revealed gene/segmental duplication in two inversion-free *sex-ratio* drive systems in *D. simulans*: Paris (Montchamp-Moreau *et al.* 2006) and Winters (Tao *et al.* 2007a).

Mendelian alleles are favored by natural selection if they increase the fitness of their carriers. However, *sex-ratio* and other alleles responsible for meiotic drive are selfish genetic elements that can spread in populations as long as their preferential transmission is not offset by strong deleterious effects. The spread of a sex-linked distorter allele causes skewed population sex ratios and triggers an evolutionary arms race at the genome scale. Selective forces favor the evolution of unlinked drive suppressors to equalize the sex ratio (*i.e.*, on the Y chromosome or the autosomes) but also favor alleles that are closely linked to the primary drive locus if they enhance distortion (Fisher 1930; Hamilton 1967). Recurrent genetic conflict over the transmission of sex chromosomes is thought to have profound evolutionary consequences, including epigenetic regulation of sex chromosomes during meiosis, genomic distribution of genes expressed in the germline, change in sex determination, and the evolution of hybrid sterility [discussed in Meiklejohn and Tao (2010) and Werren and Beukeboom (1998)]. The last hypothesis has received empirical support from studies in *Drosophila* (Phadnis and Orr 2009; Presgraves 2010; Tao *et al.* 2001). However, information about the underlying molecular mechanisms, necessary to assess the evolutionary significance of sex chromosome drive, is still critically lacking. So far, both distorter and suppressor genes together have been identified only in the Winters *sex-ratio* system of *D. simulans*, and the individual function of these genes is still elusive (Tao *et al.* 2007a; Tao *et al.* 2007b).

Here, we molecularly dissected the chromosomal region responsible for Paris *sex-ratio* drive—a textbook case in *D. simulans* (Jaenike 2008; Mercot *et al.* 1995). This system is particularly interesting in two ways. First, the etiology of drive is associated with a meiosis phenotype: the loss of Y-bearing sperm results from a disjunction failure of the Y chromosome sister chromatids during the second meiotic division (Cazemajor *et al.* 2000). Second, the emergence of Paris *sex-ratio* X chromosomes and the spread of these chromosomes in natural populations have triggered the evolution of autosomal and Y-linked suppressors (Atlan *et al.* 1997; Jutier *et al.* 2004). These features of the Paris system provide an opportunity to study the evolutionary impact of the emergence of *sex-ratio* drive and to identify a network of genes controlling segregation of the sex chromosomes.

In the Paris system, two distinct distorter elements have been fine-mapped to the cytological bands 7E-F of the *sex-ratio* reference chromosome X^SR6^: a segmental duplication and a second element located 100–150 kb away (Montchamp-Moreau *et al.* 2006). We used males carrying X^SR6^ to produce a library of bacterial artificial chromosomes (BAC). We obtained, assembled, and analyzed a sequence of about 300 kb that contains the two distorter elements. This process allowed us to identify the limits of the segmental duplication and associated repetitive elements. We were also able to shed light on the mechanism and age of the duplication event, as well as the coding potential of the different components of the duplication.

## Materials and Methods

### Fly stocks

Two types of males were used: (X^ST8^)_ST8_ males that carry the reference standard X^ST8^ chromosome and (X^SR6^)_ST8_ males that carry the reference *sex-ratio* X^SR6^ chromosome. Both X chromosomes are in the same ST8 genetic background (drive-suppressor free). To prevent recombination, the X chromosomes were maintained in the male lineage through repeated backcrosses with C(1)RM, *y*, *w* (ST8 background) females, as described in Montchamp-Moreau and Cazemajor (2002).

### BAC construction, alignment, and annotation

DNA extraction was performed on (X^SR6^)_ST8_ males. DNA was partially digested with *HindIII* and separated on a 1% agarose gel by pulse field gel electrophoresis. 27,648 BACs, each about 70 kb in length, were generated according to the protocols described in Roest Crollius *et al.* (2000). The BACs were spotted onto nylon membranes. To screen for those covering the *sex-ratio* domain previously described (Montchamp-Moreau 2006), we used ^32^P-labeled probes consisting of gene fragments scattered along the whole domain (supporting information, Figure S1). When the clones included a part of the duplication, they were sequenced for *Trf2* and/or *org-1*, for which a known polymorphism was used to discriminate between the two copies (Derome *et al.* 2008).

The BACs were sequenced by the Genoscope (Evry, France). A library was obtained for each of them after mechanical shearing of DNA and cloning of 3 kb (BAC 10c2 and 67l12) or 5 kb (BAC 58j14, 46o6, 35e19, and 24a6) fragments into a pcdna2.1 plasmid vector (Invitrogen). Additional libraries were prepared from BACs 58j14 and 46o6 by cloning 10 kb fragments into a pCNS plasmid vector (pSU18 derived). All vector DNA was purified and end-sequenced using dye terminator chemistry on ABI 3730 sequencers (Applied Biosystems, France) at ∼12× coverage. The assemblies were realized using Phred/Phrap/Consed software package (www.phrap.com; Ewing *et al.* 1998; Ewing and Green 1998; Gordon *et al.* 1998). The sequences have been deposited in the EMBL database under accession numbers FQ660547 (46o6), FQ660548 (10c2), FQ660549 (58j14), FQ660550 (35e19), FQ660551 (24a6), and FQ660552 (67l12).

The BACs were annotated using Apollo (Lewis *et al.* 2002). We performed BLAST analysis (Camacho *et al.* 2009) using the *D. melanogaster* genome as reference (http://flybase.org/, R5.29). When needed, the sequences were first aligned with ClustalW (Thompson *et al.* 2002) then manually aligned with BioEdit (Hall 1999; http://www.mbio.ncsu.edu/BioEdit/bioedit.html). The percentage of nucleotide identity between sequences was calculated using DnaSP (Librado and Rozas 2009). The repeated regions were analyzed with RepeatMasker (Chen 2004; Tarailo-Graovac and Chen 2009). Global alignment of the clones was conducted with PipMaker (Schwartz *et al.* 2000).

### Fluorescent *in situ* hybridization (FISH)

The spread of chromosomes and the hybridization were performed according to the protocol described in Montchamp-Moreau *et al.* (2006). The probe was a fragment of *Hosim1* (Figure 5 and sequence of primers in Table S1), amplified from DNA of (X^SR6^)_ST8_ males and cloned into PGEM-T Easy Vector System (Promega).

### Southern blot

High molecular weight DNA was prepared from 300 mg of adult male (Roberts 1998). Four micrograms of each extract were digested overnight with 100 U *BamHI* or 100 U *HindIII* in 200 µl final volume, precipitated after phenol/chloroform extraction, and resuspended in 30 µl TE. Overnight electrophoresis was performed on 0.7% agarose gel in TAE 1×. The transfer onto nylon membrane (Amersham Hybon-N) was performed with a Amersham VacuGene XL Vacuum Blotting System. The probe consisted of 25 ng of *Hosim1-SR* PCR product (sequence of primers in Table S1) purified with Nucleospin DNA extract II (Macherey Nagel), and then labeled with α-^32^P dCTP using High Prime DNA Labeling Kit (Roche). After a two-hour prehybridization at 68°C, the membrane was incubated overnight with the probe in 6× SSC, 5× Denhardt's reagent, 0.5% SDS, and 100 µg/ml salmon sperm DNA, then washed twice in SSC 0.2×–SDS 0.2% and twice in SSC 0.1×–SDS 0.1%.

### Quantification of DNA and cDNA by real-time PCR

The sequences of the primers are in Table S1. To estimate the copy number of *Hosim1* elements per genome, we performed six independent DNA extractions from heads of 10 males for each stock under study, using a DNeasy Tissue Kit (Qiagen). The concentration of DNA was measured with a Quant-it dsDNA HS Assay kit (Invitrogen) in a Qubit fluorometer. Quantification was performed from 5 ng of DNA with a Chromo4 thermal cycler (Bio-Rad) and Bio-Rad iQ SYBR-Green kit. The reference genes were *GAPDH* and *RPL17* (autosomal genes showing no sequence polymorphism between and within fly stocks). The efficiency of amplification was close to 100% for the six sets of primers used.

To detect and quantify *Hosim1* transcripts, the testes from two-day-old males were dissected in PBS on ice and frozen in liquid nitrogen. RNA was extracted from samples of 30 testis pairs each, using a Nucleospin RNAII kit (Macherey-Nagel) following the manufacturer's protocol. For each stock, three independent RNA extracts were obtained. Two RT-PCR reactions were performed on each extract, using Bio-Rad iScript Select cDNA synthesis kit, and 2 ng of the resulting cDNAs were used for real-time PCR. The amount of transcript was standardized to the autosomal reference genes *light* and *RPII140* that showed stable expression among samples (determined using *Genorm* (Vandesompele *et al.* 2002), *Normfinder* (Andersen *et al.* 2004), and *Bestkeeper* (Pfaffl *et al.* 2004)).

The number of DNA copies and the amount of transcript in (X^SR6^)_ST8_ males relative to (X^ST8^)_ST8_ males were estimated using the ΔΔCt method (Schefe *et al.* 2006). For each stock, the 95% confidence interval was calculated to assess the robustness and variance of our quantifications. The values were compared with a Mann-Whitney Test, using R (http://www.r-project.org/, function wilkox.test(A,B); R Development Core Team 2010).

## Results

### General organization of the *sex-ratio* region

The *sex-ratio* chromosome X^SR6^ typically leads to 90–95% female progeny in a suppressor-free background. We sequenced four overlapping BACs, named 58j14, 46o6, 35e19, and 24a6, covering 250 kb and including the candidates regions for the two distorter elements previously mapped on X^SR6^ (Figure S1, Figure S2). In addition, we partially sequenced BACs 10c2 and 67l12 to check the organization of the duplication. After assembling, we aligned the resulting sequence to the genome of *D. melanogaster* because there are numerous gaps and assembling errors in the published *D. simulans* genome. The synteny of the region appeared to be conserved between the two species (Figure S2) with 92.88% sequence identity on average.

**Figure 1:**
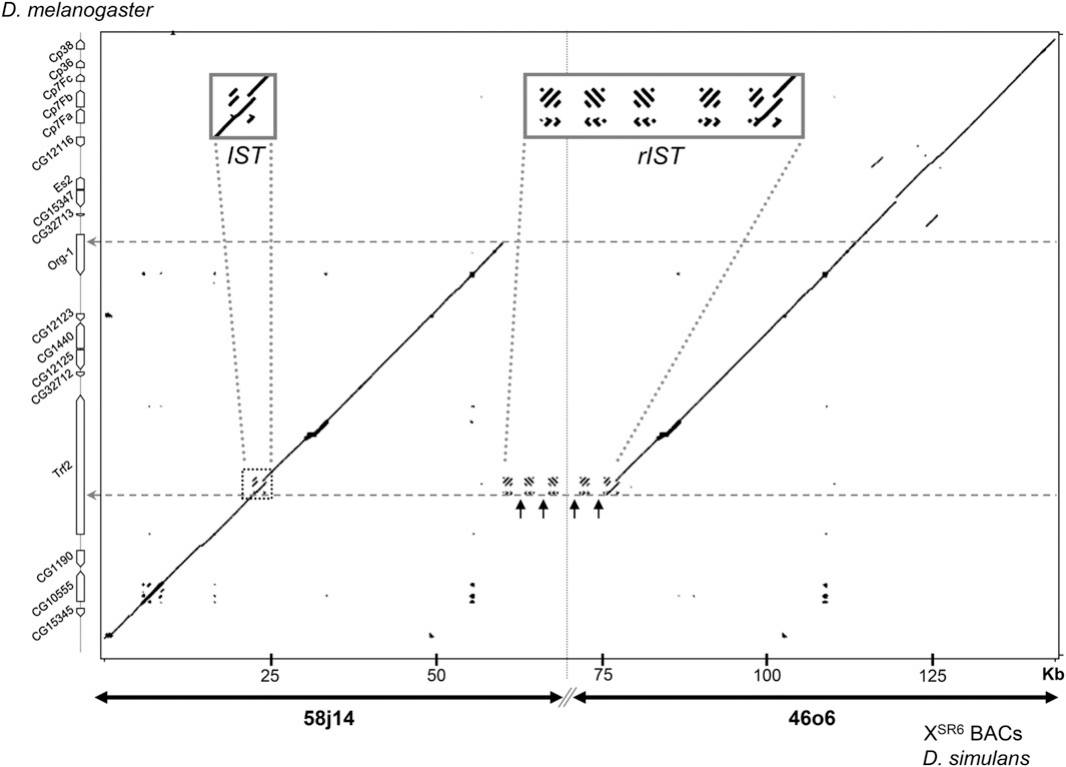
General organization of the *sex-ratio* duplication dot plot comparison of the duplication on the X^SR6^ chromosome of *D. simulans* (abscissa) with the homologous region in *D. melanogaster* (ordinate). The *D. simulans* sequence was obtained from BACs 58j14 and 46o6, which do not overlap (limits showed by the vertical dotted line). The black arrows show *Hosim1-SR* sequences (no homolog in *D. melanogaster* genome), separated by fragments with homologs in the second intron of *Trf2* (*IST*). Two horizontal dotted arrows show the limits of the duplicated fragment.

A single segmental duplication in tandem and direct orientation was detected. The duplicated fragment was found to be 37,500 bp in length (Figure 1) and contain six annotated genes. It started distally within the gene *Trf2* (second intron) and ended within the gene *org-1* (first intron). Of the four genes annotated in between, three had complete duplication: *CG12125*, *CG1440*, and *CG12123*. Analysis of their sequences did not reveal mutations that could affect their coding potential. In contrast, the distal copy of the fourth gene, *CG32712*, had a frameshift mutation caused by a 65 bp deletion within the second exon, which introduced an early stop codon.

Examination of the candidate region for the second element revealed the presence of an approximately 1 kb fragment between the genes *spirit* and *CG12065* that had no homolog in the *D. melanogaster* genome. This insertion also exists on standard X chromosomes of *D. simulans* and contains a small chromodomain-containing gene (759 bp), which is annotated in the *D. simulans* genome as *GD16106* (Figure S2). Transcripts of *GD16106* have been detected in the testis of both standard and *sex-ratio* males (D. Ogereau, unpublished data).

### Origin of the *sex-ratio* duplication

**Figure 2:**
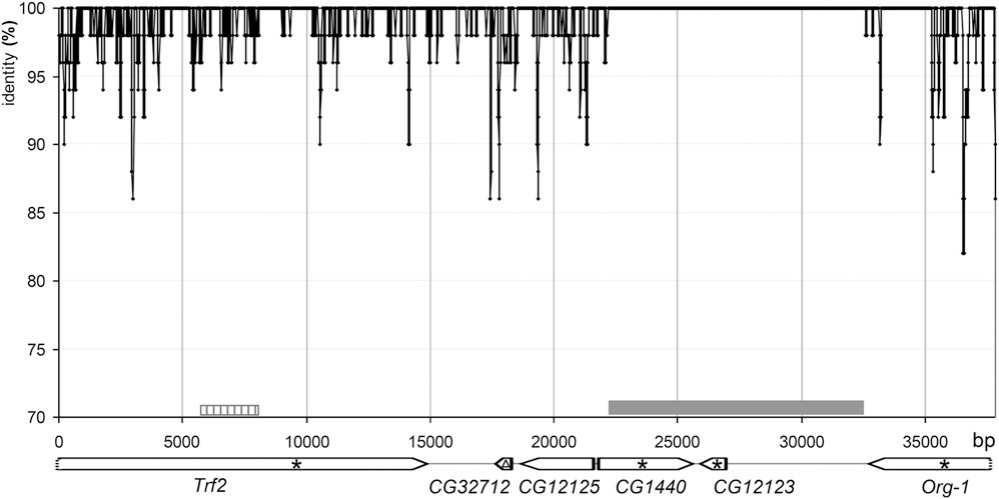
Sequence identity between the two copies of the *sex-ratio* duplication on the X^SR6^ chromosome. The analysis was performed using a 50 bp sliding window with a step size of 10 bp. The gray box represents the fragment with 100% identity; the striped box represents the region containing the DDSA traces described in Figure S3. The stars show the position of markers sequenced in the population study of Derome *et al.* (2008), and the triangle shows the position of the small deletion in the distal copy of *CG32712*.

The two copies of the duplication had a very high sequence identity score (99.49% for exons, 98.65% for introns). Figure 2 shows that nucleotide polymorphisms were not randomly scattered along the duplication: a 10,344 bp fragment was 100% identical between the two copies. This cannot be due to an assembling error because the procedure ensured that each copy of the duplication was cloned in a different BAC (see *Materials and Methods* and Figure S1). This remarkable similarity between the two copies is consistent with previous direct sequencing of markers located within the genes *CG12123* and *CG1440*, which revealed a single sequence on chromosome X^SR6^ (Montchamp-Moreau *et al.* 2006).

Outside of the identical 10,344 bp fragment, the mean identity was 98.75%, similar to the value obtained for this chromosomal region in a whole-genome analysis of polymorphism among seven independent lines of *D. simulans* (98–99%) (Begun *et al.* 2007). This suggests that the duplication event occurred in the very recent past, through production of an exact copy on the donor chromosome itself. Polymorphism was later introduced by recombination. This hypothesis is consistent with experimental evidence that revealed that recombination occurs freely between the duplication and the homologous region of standard X chromosomes (Montchamp-Moreau 2006).

**Figure 3:**
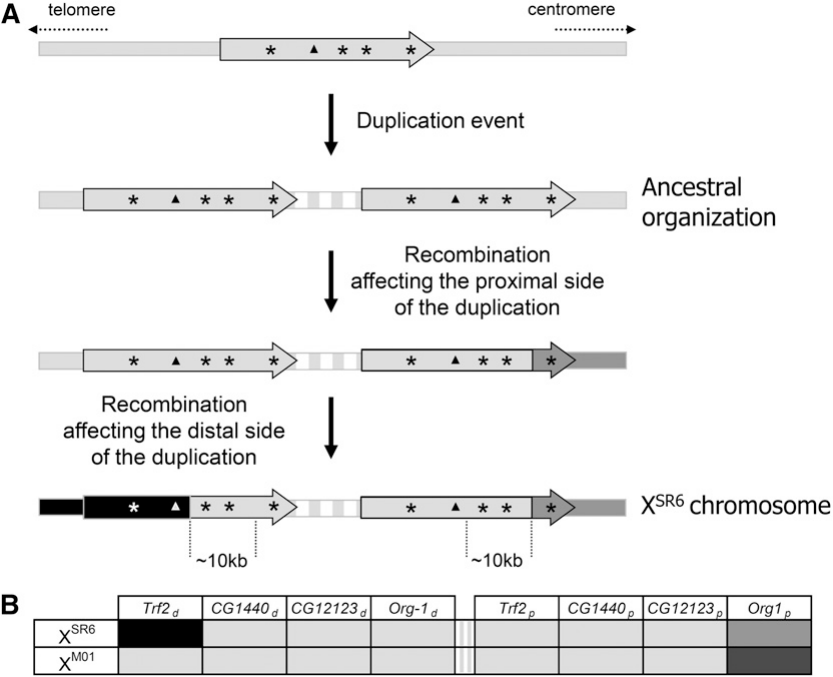
(A) Parsimonious scenario explaining the pattern of sequence variation observed between the two copies of the *sex-ratio* duplication carried by the chromosome X^SR6^. The vertical dotted lines show the limits of the 10 kb fragment with 100% sequence identity. The stars show the position of markers sequenced in Derome *et al.* (2008). The triangles show the position of *CG32712* (the white triangle stands for the deleted allele brought by recombination). The vertical gray/white strips represent the repeated motifs of the junction region. (B) Interpretation of Figure S1 in Derome *et al.* (2008). X^M01^ is a *sex-ratio* chromosome from Madagascar, carrying a combination of haplotypes commonly found there. For each marker (stars in Figure 3A), the ancestral sequence is symbolized in light gray. Alleles supposed to have been brought by recombination are in medium and dark gray (proximal recombination) and in black (distal recombination).

We therefore propose a parsimonious three-step scenario for the observed duplication pattern of X^SR6^. First was a tandem duplication of a fragment on the same chromosome (Figure 3A), followed by two recombination events, one affecting the proximal copy of the duplication and the other the distal copy. Assuming that the duplication originated recently and retained the ancestral sequence across a large portion, we should find signatures of the mechanism that generated the X^SR6^ pattern. In *D. melanogaster*, analysis of double-strand break repair (DSB) after *P*-element excision shows that DSB repair usually occurs primarily through homologous repair and, preferentially, by synthesis-dependent strand annealing (SDSA) (Engels *et al.* 1990; Nassif *et al.* 1994; Rong and Golic 2003). The template sequence is usually the allele located on the homologous chromosome or on the sister chromatid, but an ectopic site is sometimes used and thus duplicated into the DSB site (Rong and Golic 2003). The duplication-dependent strand annealing (DDSA) model is a variant of SDSA occurring after a DSB in a repeated sequence; under the DDSA model, repair uses an ectopic site that contains this repeated sequence (Fiston-Lavier *et al.* 2007).

The presence of repeated sequences at one end of the duplicated fragment suggests that the DDSA model can be applied to the *sex-ratio* segmental duplication. According to this model, instability of the DNA heteroduplex during repair leads to local dissociations of the nascent strand from the template. When reinvasion occurs within the same template, signatures of the repair mechanism can be found (Mcvey *et al.* 2004). A reinvasion upstream from the dissociation site leads to the formation of short tandem repeats within the neosynthesized copy, whereas a downstream reinvasion site that corresponds to a jump from the template causes a gap delimited by microhomology sequences within the neosynthesized copy (Fiston-Lavier *et al.* 2007). We analyzed the gaps in the alignment of the duplicated fragments carried by the X^SR6^ chromosome and, when possible, compared them to the sequences available in FlyBase (*D. simulans*, R1.3). We found five signatures of reinvasion in *Trf2*, within a fragment in which the distal copy is thought to have come from a standard chromosome via recombination (Figure 2). Three microhomologies and one tandem repeat indicated that the proximal copy was the neosynthesized sequence, whereas another tandem repeat indicated that the distal copy was the neosynthesized sequence (Figure S3). However, this latter trace can alternatively be explained by a polymerase slippage that occurred later in the proximal copy after the duplication event.

### The duplication is associated with an amplified transposable element

The domain between the two copies of the segmental duplication consists of repeated modules. Each module is composed of fragments that are homologous to fragments in the second intron of the gene *Trf2*, which we called *IST* (intronic sequence of *Trf2*), that alternate with fragments that have no homolog on the X chromosome of *D. melanogaster* (Figure 1). These fragments correspond to *Hosim1*, a class II transposable element detected in the genome of *D. simulans* and *D. sechellia* using bioinformatics methods (de Freitas Ortiz and Loreto 2009). *Hosim1* belongs to the *herves* transposable element family of the *hAT* superfamily.

**Figure 4:**
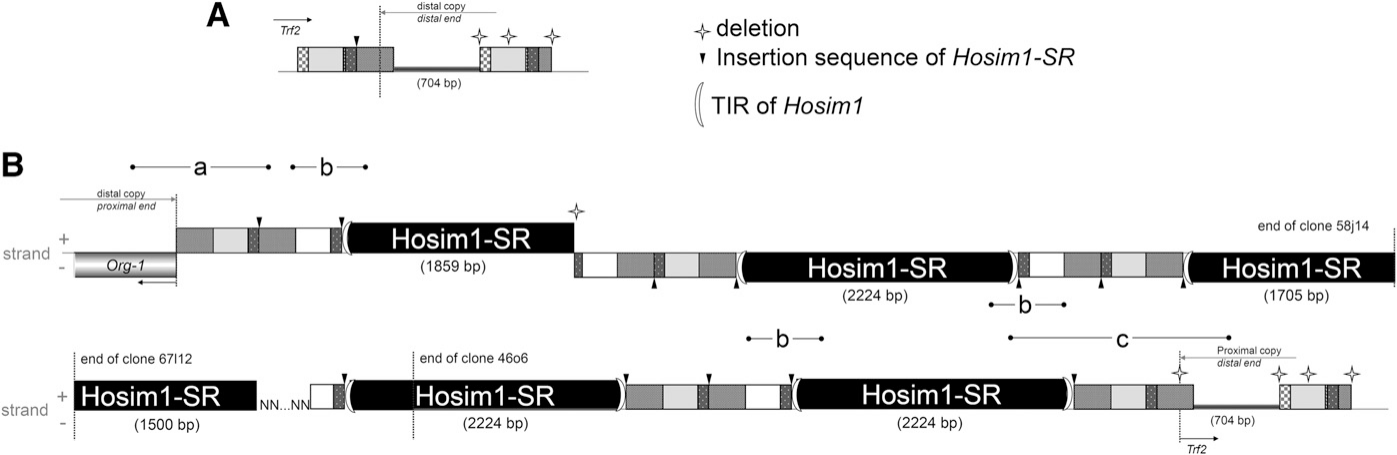
(A) Schematic representation of the canonical *IST* (Intronic Sequence of *Trf2*), found in the published *D. simulans* genome and in the distal copy of *Trf2* on chromosome X^SR6^. (B) Organization of the junction region on chromosome X^SR6^, observed in BAC 58j14 (top) and BACs 67l12 and 46o6 (bottom). It consists of alternating *Hosim1-SR* elements and direct tandems of *rIST*. Fragments (a–c) amplified by PCR to control the organization on DNA from (X^SR6^)_ST8_ males (sequence of primers in Table S1). NNN: gap in sequence assembly

In *D. simulans*, the gene *Trf2* contains two *IST* motifs located 704 bp apart (Figure 4A) that show 91.3% identity without the indels and 79.8% with the indels. This organization is conserved in *D. melanogaster* and *D. sechellia*. The motifs that alternate with *Hosim1* have been rearranged; we thus called them *rIST* (for rearranged *IST*). The *rIST*s showed more than 99% identity with each other and were always organized in direct tandems between each copy of *Hosim1*. The same 8 bp in the *rIST* sequence were duplicated at the insertion site of each *Hosim1* element.

**Figure 5:**
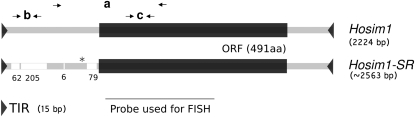
Comparison of *Hosim1-SR* with the canonical *Hosim1*: Schematic representation of the nucleotide alignment. Terminal inverted repeats (TIR): TAGTGTTGGGT. The white boxes show the position of the main deletions in *Hosim1-SR*, with their size below (in bp). The star shows the localization of the intron presented in Figure S7. (a) Position of primers used to amplify both *Hosim1* and *Hosim1-SR* transcripts (Figure 7), (b) position of primers used to estimate the number of canonical *Hosim1* (Figure 6A), (c) position of primers used to estimate the total number of elements (*Hosim1* + *Hosim1-SR*, Figure 6A), and the total amount of transcripts (Figure 6B).

There was 100% identity among the *Hosim1* copies associated with the duplication (excepted for a deletion of the 3′ part of the first element in the 58j14 clone). This finding suggests either that their amplification is very recent or that genetic conversion is frequent at this locus. We called these copies *Hosim1-SR*, because they were noticeably different from the four *Hosim1* forms already annotated in the *D. simulans* genome [accession number CH986553, CH981769, CM000363, CH982471 (partial sequence)]. While the four canonical forms differ from each other by only 23 SNPs and a poly-T stretch, *Hosim1-SR* had four deletions in the 5′ noncoding region (Figure 5) and differed from the canonical forms by 28 SNPs. These differences, however, do not affect the size of the transposase predicted by *ORF Finder* (Rombel *et al.* 2002). Amino acid alignment with *Hermes* transposase showed that both *Hosim1* and *Hosim1-SR* retained the DDE amino acids involved in the enzyme’s function (Perez *et al.* 2005). Furthermore, both *Hosim1* and *Hosim1-SR* contain the LDPR sequence that is characteristic of the majority of *hAT* transposable elements (Handler and Gomez 1997). The terminal inverted repeats (TIR) of *Hosim1* are conserved in *Hosim1-SR*.

While potentially active *Hosim1*-like elements (*Hosec1)* have been described in the genome of *D. sechellia* (de Freitas Ortiz and Loreto 2009), we found only one element, incomplete and diverging, in the *D. melanogaster* genome. Figure S4 shows a maximum likelihood tree obtained from the published sequences.

### Checking the organization of the duplication

First, we confirmed that the presence and amplification of *Hosim1-SR* at the junction of the duplicated segments was not due to a cloning artifact. We performed fluorescent *in situ* hybridization (FISH) on polytene chromosomes with a probe targeting both *Hosim1* and *Hosim1-SR* (Figure 5). In standard males (X^ST8^)_ST8_ we detected two hybridization sites, on the chromosomal arms 3L (80A) and 2L (42C) (Figure S5). These sites correspond to the *Hosim1* copies identified in the published *D. simulans* genome (accession number: CM000363 and CH986553, respectively). In (X^SR6^)_ST8_ males, which carry the *sex-ratio* X^SR6^ chromosome in the same autosomal background as (X^ST8^)_ST8_ males, we observed an additional site in the cytological band 7E of the X chromosome. Thus, this extra signal colocalizes with the *sex-ratio* duplication (Montchamp-Moreau *et al.* 2006).

Then, because of the potential impact on the expression level of neighboring genes, we checked the gene organization at the limits between the duplicated fragments and the intervening repeated region. We extracted DNA from (X^SR6^)_ST8_ males, and we used PCR to amplify fragments that overlap between *org-1* and *Hosim1-SR* and those that overlap between *Hosim1-SR* and *Trf2* (Figure 4B, a–c). The sequences were found to be identical to those in the BACs.

### Size and organization of the junction region

To check the organization of the junction region, we performed further screening of the BACs library and found two additional BACs (10c2 and 67l12, Figure S1) that contain a larger part of the junction region. We found no BAC that encompasses the whole region (*i.e.*, that contains the adjacent end of the segmental duplication on both sides). The abundance of repeated motifs with very high similarity and rearrangements in clones made it impossible to completely sequence and assemble BACs 10c2 and 67l12. Nevertheless, we found only *Hosim1-SR* and *rIST* sequences in this region. The partial sequence assembly and the digestion of BACs 10c2 and 67l12 by *HindIII* (D. Ogereau, unpublished data) confirmed the organization proposed in Figure 4 and indicated that the junction region in 67l12 contains only *Hosim1-SR*/*rIST*/*rIST* modules.

In addition, we performed Southern blots using high molecular weight genomic DNA and hybridization with a *Hosim1-SR* probe (Figure S6). *HindIII* digest produced bands shared by (X^ST8^)_ST8_ and (X^SR6^)_ST8_ males, which likely correspond to autosomal copies. A restriction site is present in the 5′ region of *Hosim1-SR* but not in *IST* or *rIST* sequences (Figure S6B). Because (X^SR6^)_ST8_ males produced a strong specific band of ∼3.6 kb, it follows that the junction region contained mainly, if not exclusively, a succession of *Hosim1-SR*/*rIST*/*rIST* modules in the same orientation as found in clones 46o6 and 67l12. However, a light band of ∼4.3 kb was also observed, which should correspond to a module in the opposite orientation, as found in clone 58j14 (Figure 4). This could be the signature of sporadic rearrangements, possibly favored by the repetitive structure.

### Estimating the number of *Hosim1* in (X^SR6^)_ST8_ males

According to the data provided by the BACs, the junction region contains at least six copies of *Hosim1-SR* (Figure 4). We also estimated directly on the X^SR6^ chromosome the total size of the repeated region using high molecular weight genomic DNA digested by *BamHI*. There is no *BamHI* restriction site in *rIST* and *Hosim1-SR*: the closest sites on either side of the junction domain are in the distal copy of *org-1* (∼1.6 kb apart) and in the 5th exon of the proximal copy of *Trf2* (∼10.1 kb apart). Hybridization of the Southern blot with a probe spanning the whole *Hosim1-SR* element revealed a large fragment estimated at 26–36 kb, which corresponds to 4.1–6.9 copies of *Hosim1-SR*/*rIST*/*rIST* modules of ∼3.6 kb (Figure S6).

**Figure 6:**
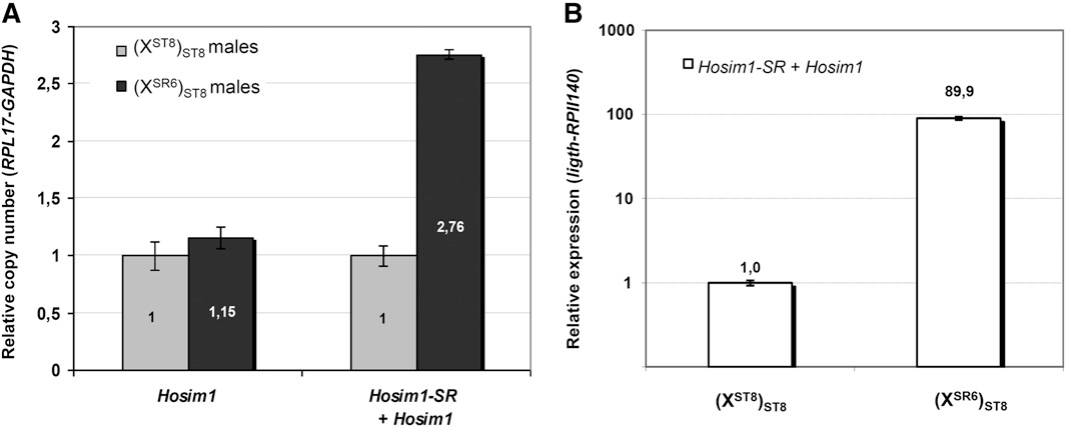
Quantification of *Hosim1* copy number (A) and *Hosim1* transcripts (B) by real-time PCR. The values in (X^SR6^)_ST8_ males were estimated relative to that in (X^ST8^)_ST8_ males. Vertical bars: confidence interval (95%). (A, left) “Canonical” elements (see text). (A, right) Canonical elements plus *Hosim1-SR*. The reference genes were *RPL17* and *GAPDH*. (B) Total amount of testicular transcripts (= *Hosim1* + *Hosim1-SR*). Reference genes were *light* and *RPII140*.

We used real-time PCR to obtain an independent estimate of the number of transposable elements in the duplication. Again we used (X^SR6^)_ST8_ and (X^ST8^)_ST8_ males, which differ only by the X chromosomes. First, the published *Hosim1* form (de Freitas Ortiz and Loreto 2009) was specifically amplified using primers designed within the region deleted in *Hosim1-SR* [Figure 5,(b)] We observed a weak difference between the two types of males (1.15 times more copies in (X^SR6^)_ST8_ than in (X^ST8^)_ST8_; *P* = 0.04), suggesting that there is no extra canonical *Hosim1* on the X^SR6^ chromosome (Figure 6A). We quantified the total number of elements, *Hosim1* plus *Hosim1-SR*, by amplifying a sequence located in the coding region shared by the two forms [Figure 5,(c)]. We found 2.76 times more copies in (X^SR6^)_ST8_ than in (X^ST8^)_ST8_ males (*P* = 2.4 × 10^−12^, Figure 6A). According to the results of the FISH experiment (Figure S5), there should be four copies of *Hosim1* in the autosomal genome shared by (X^ST8^)_ST8_ and (X^SR6^)_ST8_ males (a single and homozygous copy at each autosomal site). Under this hypothesis, about 11 copies must be present in (X^SR6^)_ST8_ males and, consequently, seven copies on the X^SR6^ chromosome [confidence interval 95% (6.85–7.19)].

### Expression of *Hosim1* and *IST* in (X^ST8^)_ST8_ and (X^SR6^)_ST8_ males

**Figure 7:**
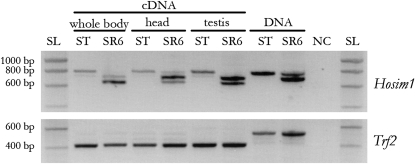
Detection of *Hosim1* transcripts by RT-PCR. The primers used straddle the deletion of 79 bp specific of *Hosim1-SR* [Figure 5,(a)], so this element must produce a shorter band (699 bp) than the canonical *Hosim1* (784 bp). Even shorter fragments were obtained from cDNAs revealing an intron of 67nt (see text). Amplification of *Trf2* with primers straddling an intron was used to control the lack of DNA contamination in the cDNA samples. NC, negative control (no cDNA nor DNA); SL, SmartLadder DNA ladder (Eurogentec); SR6, (X^SR6^)_ST8_ males; ST, (X^ST8^)_ST8_ males.

To determine whether *Hosim1* and, in particular, the *Hosim1-SR* form are still active, we performed PCR on cDNA with a primer pair straddling the deletion characteristic of the *Hosim1-SR* form [Figure 5,(a)]. Transcripts were present in the whole body, in head, and in testes of both *sex-ratio* and standard males. We found that both forms were expressed in (X^SR6^)_ST8_ males and that the cDNA fragments were shorter than DNA fragments (Figure 7). Sequencing the shorter form revealed a 67 bp intron associated with *Hosim1-SR* (Figure S7). The splicing occurred for *Hosim1* transcripts, too, but appeared to be less efficient [see (X^ST8^)_ST8_ males in Figure 7]. In (X^SR6^)_ST8_ males, the splicing seemed to be more efficient in the testes. Real-time PCR showed that the total amount of testicular transcripts was about 90 times higher in (X^SR6^)_ST8_ males than in (X^ST8^)_ST8_ males (Mann-Whitney test, *P* = 2.2 × 10^−16^, Figure 6B).

**Figure 8:**
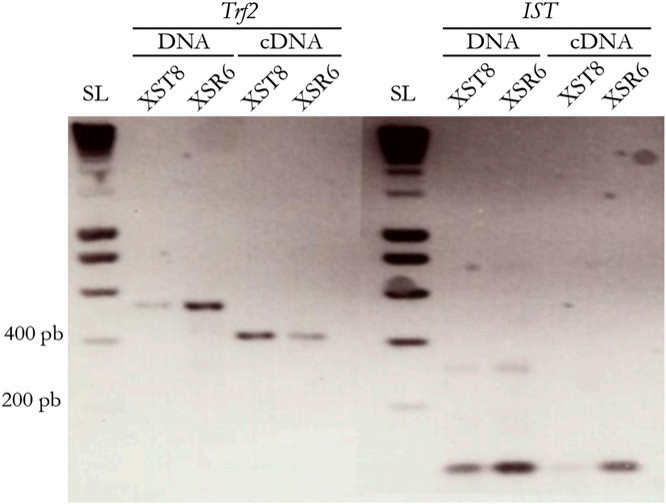
Detection of *IST* transcripts by RT-PCR. The *IST* and *rIST* probes were designed within the region shown in light gray in Figure 4. Amplification of *Trf2* gene marker was used to control the lack of DNA in the cDNA samples (see Figure 7). SL, SmartLadder DNA ladder (Eurogentec); XSR6, (X^SR6^)_ST8_ males; XST8, (X^ST8^)_ST8_ males.

To test for the presence of transcripts that contain the *IST* or *rIST* sequences (light gray boxes in Figure 4), we designed primers that amplify both forms (Table S1); these noncoding motifs appeared to be transcribed, and more cDNAs were detected in (X^SR6^)_ST8_ males (Figure 8) than in (X^ST8^)_ST8_.

## Discussion

Here we provide evidence that the X^SR6^ chromosome carries a recent tandem segmental duplication of 37.5 kb that originated through the production of an exact copy on the donor chromosome itself and that changed the copy number of six genes. By contrast, the second element required for drive is not associated with rearrangement. Yet, in the candidate region, we noticed a small gene (*GD16106*) that does not exist in the *D. melanogaster* genome. The molecular data allowed us to propose a mechanism for how the duplication was generated and to retrace its history.

### Characteristics of the duplication and possible mechanisms

The two copies of the duplicated chromosome fragment are separated by repeated modules, each of which contains a *Hosim1* transposable element that has small deletions but that is potentially active, and tandem motifs derived from an intronic sequence of *Trf2* (*rIST*). Amplification of the modules may be responsible for the additional dense band revealed after DAPI coloration in the 7E section on the X^SR6^ polytene chromosome (Montchamp-Moreau *et al.* 2006), which reflects a local modification of the chromatin structure. The highly repeated nature of this region prevented its complete sequencing and assembly, but three independent methods indicated that the X^SR6^ chromosome carries six or seven modules. Organization like this is a potential source of instability and unequal crossovers; this instability likely produces variation in the number and organization of motifs among natural *sex-ratio* X chromosomes.

About 25% of the tandem duplications detected in the genome of *D. melanogaster* show at least one repetitive element at the breakpoint (Fiston-Lavier *et al.* 2007). The local sequence organization, with two repeated modules 704 bp apart within *Trf2*, could have favored a double-strand DNA break. Alternatively, the transposable element may have generated the break; indeed *Hosim1* is a class II transposable element that is mobilized by a DNA intermediate through a ‘‘cut-and-paste’’ mechanism (de Freitas Ortiz and Loreto 2009). According to the DDSA model (Fiston-Lavier *et al.* 2007), a double-strand break within a repeated sequence (here *IST* or *Hosim1*) is repaired via homologous–base pairing using another copy of this repeated sequence as template. The repair leaves specific signatures that were detected on the X^SR6^ chromosomes and that allowed us to identify the proximal copy of the duplication as the neosynthesized sequence. The organization of the junction domain between the duplicated fragments probably resulted from secondary amplification of repeated sequences.

### The duplication should induce quantitative and qualitative changes in transcripts

Testicular transcripts of the three fully duplicated genes *CG12125*, *CG1440*, and *CG12123* had been detected using rtPCR in (X^SR6^)_ST8_ males, and a polymorphism in cDNA sequences led to the inference that both copies of *CG12125* are active (Montchamp-Moreau *et al.* 2006). It was not possible to determine whether both copies of *CG12123* and *CG1440* are active because the distal and proximal copies are 100% identical. As none of the three genes on the X^SR6^ chromosome revealed any trace of frameshift or stop mutations, their duplication should result in quantitative changes in canonical transcripts. cDNA sequencing revealed that both copies of *CG32712* are expressed in (X^SR6^)_ST8_ males (D. Ogereau, unpublished data). However, the 65 bp deletion in the second exon of the distal copy of *CG32712* causes a nonsense mutation, so the associated mRNA cannot produce functional proteins. Other *sex-ratio* X chromosomes (*e.g.*, X^M01^ depicted in Figure 3B) have been found to carry two 100% identical copies of the complete, likely original, proximal copy of X^SR6^. Thus, the deleted allele must have been introduced by recombination. As X^SR6^ shows strong drive ability, this suggests that *CG32712* is not the distorter element in the duplication.

Although *Trf2* and *org-1* are not fully duplicated, transcripts produced by both copies of each of these genes were found in the testis of (X^SR6^)_ST8_ males (Montchamp-Moreau *et al.* 2006). In the *D. melanogaster* subgroup, about 78% of new genes have arisen from duplications, and of these, 32% formed chimerical structures by recruiting flanking sequences into their coding region (Zhang *et al.* 2008). Located on either side of the junction domain of the *sex-ratio* duplication, the 5′ deleted copies of the *Trf2* and *org-1* genes are potential actors of such a process. The distal copy of *org-1* lacks its first exon, which contains the start codon and the first 54 amino acids. The nearest *Hosim1-SR* element in the junction domain is in the opposite orientation (Figure 4B), suggesting that a chimerical transcript cannot be produced. *Trf2* is more complex because two different *Trf2* transcripts have been reported. Kopytova *et al.* (2006) described a long *Trf2* transcript (∼7.6 kb), thought to produce two proteins, one of 175 kD and one of 75 kD, that differed in their N-terminal domain; the shorter protein could have been produced via an internal ribosome entry site (IRES). Short transcripts (∼3.9 kb), initially described by Rabenstein *et al.* (1999), can only produce the shorter protein. In the X^SR6^ chromosome, the proximal copy of *Trf2* lacks the two first exons of the long transcript described by Kopytova *et al.* (2006). However, it could potentially produce the short transcript described by Rabenstein *et al.* (1999), and it retains the complete coding sequence for both proteins.

The repeated sequences (*Hosim1* and *rIST*) amplified in the junction region appeared well expressed in (X^SR6^)_ST8_ males: *Hosim1* transcripts were found to be about 90 times more abundant in the testis of (X^SR6^)_ST8_ males than in (X^ST8^)_ST8_ males (Figure 6B). As there are only 2.7 more *Hosim1* copies in the genome of the (X^SR6^)_ST8_ males than in (X^ST8^)_ST8_ males, either the *Hosim1-SR* form is expressed much more in the testis than the autosomal forms or there is a general overexpression of *Hosim1* elements in *sex-ratio* males. We also detected cDNA containing *IST*/*rIST* sequences, although they are certainly noncoding (they are intronic sequences, and bioinformatics software did not detect any ORF). Such noncoding RNAs can be involved in a variety of processes, including dosage compensation, posttranscriptional gene silencing, regulation of transposable elements, and chromatin remodeling (Van Wolfswinkel and Ketting 2010). Because some *rIST*s and the 5′ deleted proximal copy of *Trf2* are in the same orientation (Figure 4B), together they might produce chimerical transcripts.

### Age of the duplication and evolutionary prospects

The 10,344 bp fragment of the *sex-ratio* duplication on the X^SR6^ chromosome with 100% identity between the copies (Figure 2) is too long to have arisen by gene conversion. We thus assumed that it represents the ancestral state, and no recombination with a standard X occurred in this region. This allows us to estimate the age of the duplication, assuming a conservative value of ∼2 × 10^−8^ recombination/bp/generation in the region (Derome *et al.* 2008; Derome *et al.* 2004; Montchamp-Moreau *et al.* 2006). As the probability of recombination or mutation is low, the number of these events follows a Poisson distribution (Sawyer and Hartl 1992). The probability of two copies of a fragment of size *L* remaining fully identical is given by the formula *e^-2(r+µ)Lt^*, where *t* = number of generations (10 per year) and *µ* = mutation rate/bp/generation [µ = 10^−8^ (Rozas *et al.* 2001)]. It follows that the duplication event likely took place less than 483 years ago (*P* = 0.05). That the duplication is so recent is well supported by previous molecular population genetics studies (Bastide *et al.* 2011; Derome *et al.* 2008). First, among the four marker loci that were surveyed, there was no fixed difference between the duplicated X^SR^ and standard X^ST^ chromosomes sampled in the wild. In addition, these previous studies showed that most of the X^SR^ chromosomes collected in Madagascar only 10 years ago still carried the presumed ancestral sequence with no trace of even a singleton mutation at these marker loci. The predominant variant found in this population could even have retained an ancestral fragment of larger size than that in X^SR6^ (Figure 3B). Thus, the structure of X^SR6^ is not exceptional among the present distorter X chromosomes. Note also that the region in between the identical 10,344 bp fragments may not have undergone recombination at all. If this is the case, then the duplication is younger than estimated above. *Sex-ratio* X chromosomes, however, can reach high frequencies in natural populations (≥50%) (Atlan *et al.* 1997; Jutier *et al.* 2004). In that case, the probability of recombination occurring between X^SR^ and X^ST^ chromosomes would be lower than the overall recombination rate for the genome region, thus the duplication age could be more than 483 years.

Segmental duplications are frequent on the X chromosome of *D. melanogaster*, but only 7.21% of them are more than 10 kb long. In addition, tandem duplications are almost always shorter than other duplications (Fiston-Lavier *et al.* 2007). This makes the *sex-ratio* duplication an exceptional event with regard to both its size (more than 37 kb) and its gene content. This kind of duplication is probably deleterious most of the time and, thus, destined to disappear quickly. In the present case, the duplication has a strong, negative effect on male fertility that is a direct consequence of drive (Angelard *et al.* 2008; Atlan *et al.* 2003; Atlan *et al.* 2004) and should cause many other perturbations via overexpression of the six duplicated genes or *rIST* and *Hosim1-SR* activity. In this respect, meiotic drive can be understood as a process that allows genetic rearrangements, such as duplications or inversions, to be maintained in the genome in spite of associated deleterious effects, as first proposed by Hedrick (1981). These rearrangements can persist for extended evolutionary periods, as demonstrated by one of the inversions associated with the *sex-ratio* trait in *D. pseudoobscura* with an apparent divergence time of about 1 million years (Babcock and Anderson 1996; Kovacevic and Schaeffer 2000). This allows time for the genetic innovation to coevolve with the host genome and eventually lead to a neutral or advantageous form.

Now that the sequence of the reference chromosome X^SR6^ is known, precise study of gene expression is the next step in understanding the link between the duplication and *sex-ratio* meiotic drive. The duplication affects the copy number of six genes and is associated with several copies of an active transposable element and repeated modules that produce noncoding RNAs. Therefore, we must determine which of these components is involved in *sex-ratio* drive. In this respect, the duplicated X^SR6^ sequence will be a precious tool for analyzing polymorphism along this region among natural distorter chromosomes, with the goal of identifying a correlation between drive ability and duplication structure. It will also allow for the development of appropriate transgene constructs for the functional validation of candidate genes or sequences. Unraveling the molecular mechanisms that underlie the Paris *sex-ratio* drive should help us understand the evolutionary significance of segregation distorters.

## Supplementary Material

Supporting Information
