# Supplementary material for: Sequence Analysis of the Segmental Duplication Responsible for Paris Sex-Ratio Drive in Drosophila simulans
Source: G3 (Bethesda). 2011 Oct 1;1(5):401–10. doi: 10.1534/g3.111.000315 (PMC3276153; doi:10.1534/g3.111.000315)
Supplement: Supporting Information [file supp_1_5_401__index.html]

Supporting Information 

# Sequence Analysis of the Segmental Duplication Responsible for Paris *Sex-Ratio* Drive in *Drosophila simulans*

## Supporting Information for Fouvry *et al.*, 2011

**Files in this Data Supplement:**

- Supporting Information - Figures S1-S7 and Table S1 (PDF, 3.3 MB)
- Figure S1 - Selection of the clones used to sequence the *sex-ratio* region on XSR6 (PDF, 108 KB)
- Figure S2 - Dot plot comparison of the *sex-ratio* region on the XSR6 chromosome of *D. simulans* with the homologous region in *D. melanogaster* (PDF, 224 KB)
- Figure S3 - Signatures of the DDSA model (PDF, 1.2 MB)
- Figure S4 - Maximum likelihood tree of *Hosim1* homologous sequences (HKY85 model) rooted with *D. melanogaster sequence* (*dmel*)(PDF, 68 KB)
- Figure S5 - Visualization of *Hosim1* insertion sites by *in situ* hybridization on polytene chromosomes (PDF, 628 KB)
- Figure S6 - Control of the organization of the junction region (PDF, 508 KB)
- Figure S7 - Identification of an intron in *Hosim1-SR* (PDF, 548 KB)
- Table S1 - PCR primers used in the study (PDF, 52 KB)
