# Supplementary material for: Sequence Analysis of the Segmental Duplication Responsible for Paris Sex-Ratio Drive in Drosophila simulans
Source: G3 (Bethesda). 2011 Oct 1;1(5):401–10. doi: 10.1534/g3.111.000315 (PMC3276153; doi:10.1534/g3.111.000315)
Supplement: Supporting Information [file supp_1.5.401_000315.pdf]

A

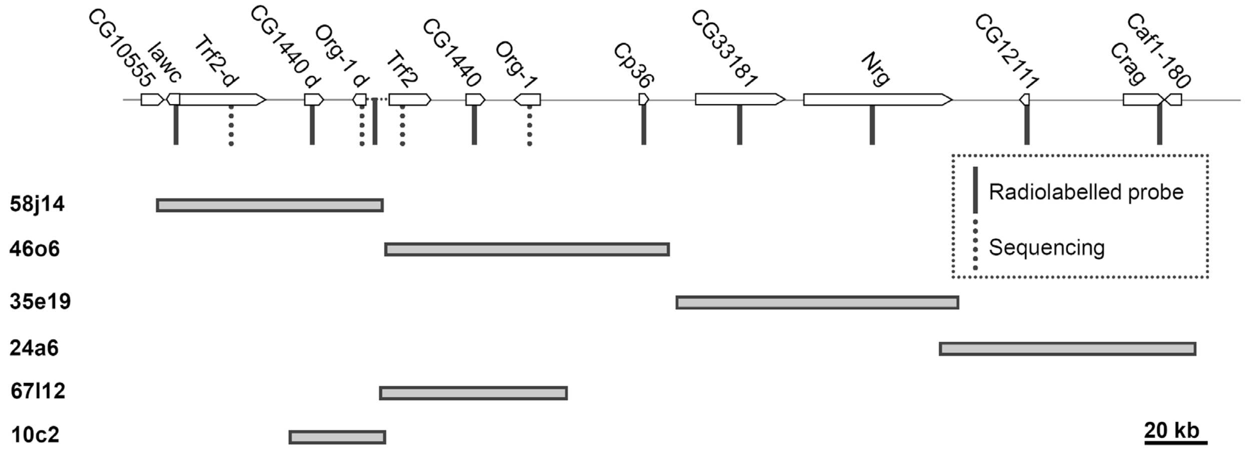

B

Locus	Name	Primers	Size bp
		Radiolabelled probes	
lawc	DMCG32711ex1-1<br>DMCG32711_1_5	TGTGTGAGTCGCGATAAACG<br>TCAGCTCTGGTAGCGAAACA	738
CG1440	DMCG1440ex35-1<br>DMCG1440ex35-2	GCGTTATGTTAAGCCGCATT<br>GGCGTTCATCACCAGATAGC	598
IST-Trf2	inter43_44-1<br>Inter43_44-2	AACCGGTTATGATGCGAAAG<br>CCGTATAGCTCAACCGATTTG	936
Cp36	CP36-F1<br>CP36-R1	CAGACAGCGAGCAGTAGACG<br>GTGGAGCCTCGATCTTCTTG	802
CG33181	DMCG33181-3<br>DMCG33181-4	TTCTACGGAATGCAAACGTG<br>TTGAGCGATACAATCGAACG	694
Nrg	NRGintron1F<br>NRGintron1R	GCAGTGCAGAAAGCAAAGGT<br>CCGGTTGTTTGGTGATTCTT	350
CG12111	DSC12111-F1<br>DSC12111-R1	TCTGCTGTTAATCCTGACGA<br>TACCACTTAATGTAGGTGAACTTGA	614
Crag	DMCRAG7<br>DMCRAG8	GTACAGATCGAACGGCAGGT<br>CATACTCGCGATGGAATGTG	805
		Sequencing probes	
Trf2	DMTRF2-1<br>DMTRF2-2	GCCATCCATACACCACTGC<br>GAACGTTGCTTGCGGAAA	530
Org-1	OrgF1<br>OrgR1	AGCCACTTTCGCACTTTCAT<br>GTGCCATCACATCCCGAAAG	1054

Figure S1 Selection of the clones used to sequence the *sex-ratio* region on X^{SR6}. (A): Schematic representation of the *sex-ratio* region on the X chromosome and position of the clones. For the sake of simplicity, we only represented the genes used to design probes for the selection of clones and two genes adjacent to the region under study. The open boxes represent the genes. The grey boxes represent the clones, with their names on the left. The vertical bars show the fragments used as probes to position the clones on the X chromosome (see materials and methods), the dotted vertical lines represent the fragments that were sequenced to discriminate between the proximal and distal copies of the duplication. (B): PCR primers to produce the fragments used as probes (ordered from left to right).

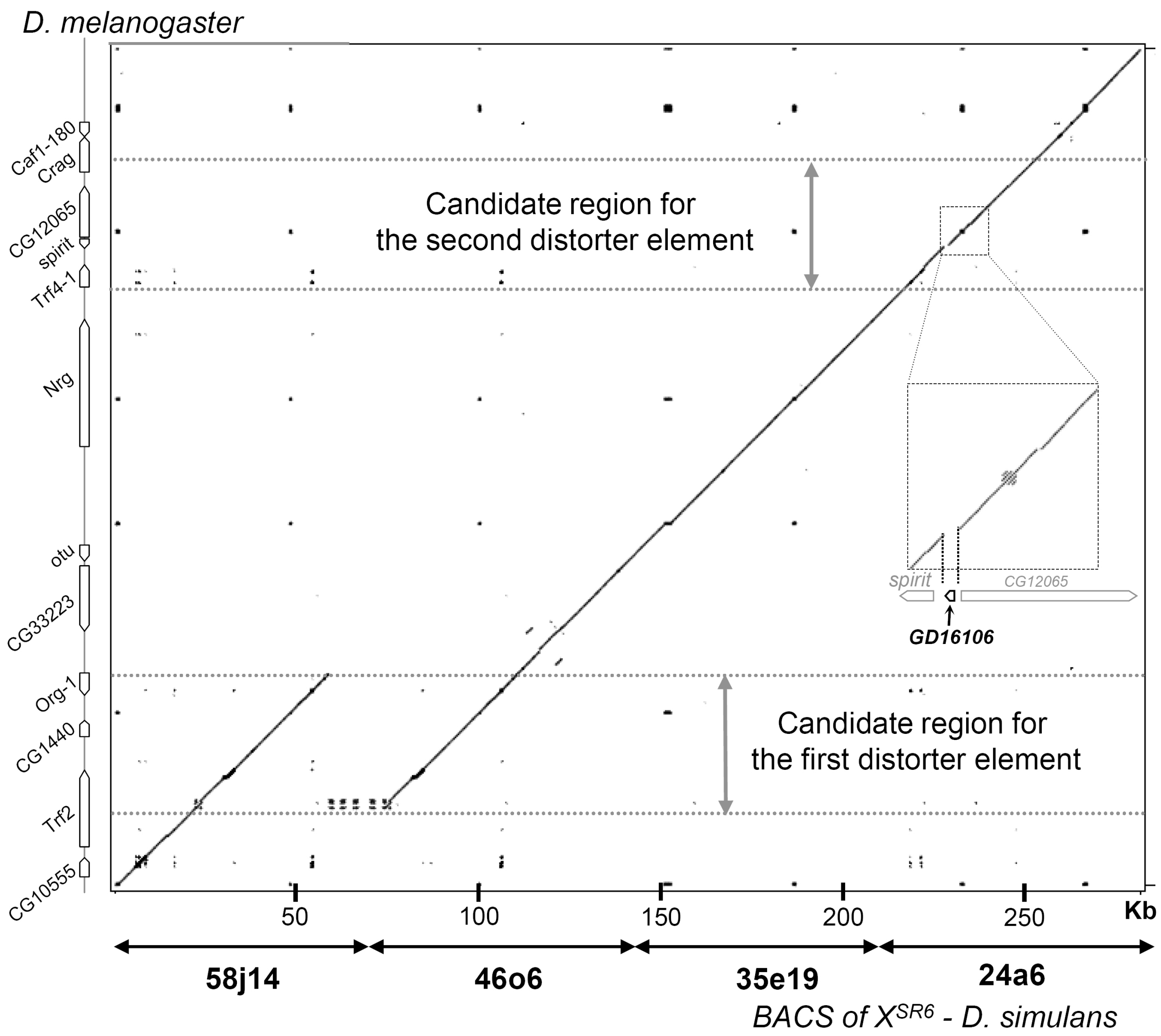

Figure S2 Dot plot comparison of the *sex-ratio* region on the X^{SR6} chromosome of *D. simulans* (below) with the homologous region in *D. melanogaster* (left). Boxes along the vertical axis represent the genes annotated in *D. melanogaster* genome. The limits of the candidate region for the second element involved in drive are those determined in Montchamp-Moreau *et al* (2006).

DDSA traces interpretation			
**Site**	**Position**	**DDSA type of trace**	**Deduction of the neo-synthesized copy**
1	5838	Tandem repeat	Proximal copy (4606)
2	5949	Micro-homology traces	Proximal copy (4606)
3	6198	Tandem repeat	Distal copy (58j14)
4	6992	Micro-homology traces	Proximal copy (4606)
5	8787	Micro-homology traces	Proximal copy (4606)

Legends:
tandem repeat
**micro-homology traces**

Site 1: Position: 5838 bp

```
XSR6d-58j14   CCGGCATTTATCAAAACGGCAATGTCG---------GTGGAATCTTTAACTAGAAACAAC
XSR6p-46o6    CCGGCATTTATCAAAACGGCAATGTCGTGGAATCGTGTGGAATCTTTAACTAGAAACAAC
              ***************************          ************************

Dsim          CCGGCATTTATCAAAACGGCAATGTCG---------GTGGAATCTTTAACTAGAAACAAC
Dsec          CCGGCATTTATCAAAACGGCAATGTCG---------GTGGAATCTTTAACTAGAAACAAC
Dmel          GAGGCATTTATCAAATCGGCAATGTCG---------GGGGAATCTTCAACTAGAAACAAC
```

Site 2: Position: 5949 bp

```
XSR6d-58j14   ATAGATCTCTTTGGCATTAAAT------GAGTGAAGCTCCTAAATGTACATATGCAATAT
XSR6p-46o6    ATAGATCTCTTTGGC----------------------TCCTAAATGTACATATGCAATAT
              ***************                      ***********************

Dsim          ATAGATCTCTTTGGCATTAAAT------GAGTGAAGCTCCTAAATGTACATATGCAATAT
Dsec          ATAGATCTCTTTGGCATTAAATTAAAATGAGTGAAGCTCTTAAATGTACATATGCAATAT
Dmel          ATAGATCTTTTTGGCATTAAAT------GAGTGAAGCTCCTAAACATACATATGCAATAT
Dyak          ATAGACCTTTTTGGTATTAAAT------GAGAGAAGCTCCTAAATGTACATTTGAAATAT
```

Site 3: Position: 6198 bp

```
XSR6d-58j14   TCGATGTTGGTGCATAACTGTTGTTGTTGCATAACTGTTGTTGTTGCTGTTGCTCGATTT
XSR6p-46o6    TCGATGTTGGTGCATAACTGTTGTTGTTGC-----------------TGTTGCTCGATTT
              ******************************                 *************

Dsec          TCGATGTTGGTGCATAACTGTTGTTGTTGC-----------------TGTTGCTCGATTT
Dsim          TCGATGTTGGTGCATAACTGTTGTTGTTGC-----------------TGTTGCTCGATTT
Dmel          TCGATGTTGGTGCATAACTGTTGTTGTTG--------------------TTGCTTGATTT
```

Site 4: Position: 6992 bp

```
XSR6d-58j14   AATTTGCGTGTTCTTATTTTCCCAAATGTCAACTTTTCCAATTATTTATTTACTTTTTAT
XSR6p-46o6    AATTTACGTGTTCTTATTTTCCCAA----------------TTATTTATTTACTTTTTAT
              ***** *******************                *******************

Dsim-a        AATTTGCGTGTTCTTATTTTCCCAAATTTCAACTTTTCCAATTATTTATTTACTTTTTAT
Dsim-b        AATTTGCGTGTTCTTATTTTCCCAAATGTCAACTTTTCTAATTATTTATTTACTTTTTAT
Dsec          AATTTGCATGTTCTTATTTTCCCAAATGTCAACTTTTCCGATTATTTATTTACTTTTGAT
Dmel          AATTTGCATGTTTTCATTTTCCCAAATGTCAACTTTACGA-----TTATTAACTTTTATT
```

Site 5: Position: 8787 bp

```
XSR6d-58j14   GAGTACCAGGATTCAAATCACGAGTACCAGGACTTGAATCACGAGTATCAGGACTCAACA
XSR6p-46o6    GAGTACCAGGATTCAA---------------------ATCACGAGTATCAGGACTCAACA
              ****************                     ***********************

Dsim          GAGTACCAGGATTCAAATCACGAGTACCAGGACTTGAATCACGAGTATCAGGACTCAACA
```

Figure S3 Signatures of the DDSA model. Several traces of the DDSA model have been detected when aligning the duplicated fragments according to Fiston-Lavier *et al* (2007): XSR6d-58j14 = distal copy on X^{SR6}, XSR6p-58j14 = proximal copy on X^{SR6}. The stars show the position identical between the copies. Homologous sequences in other species were found in flybase. Dsim: *D. simulans*, Dsec: *D. sechellia*, Dmel: *D. melanogaster*, and *Dyak*: *D. yakuba.* The positions of sites correspond to those in abscissa of Figure 2.

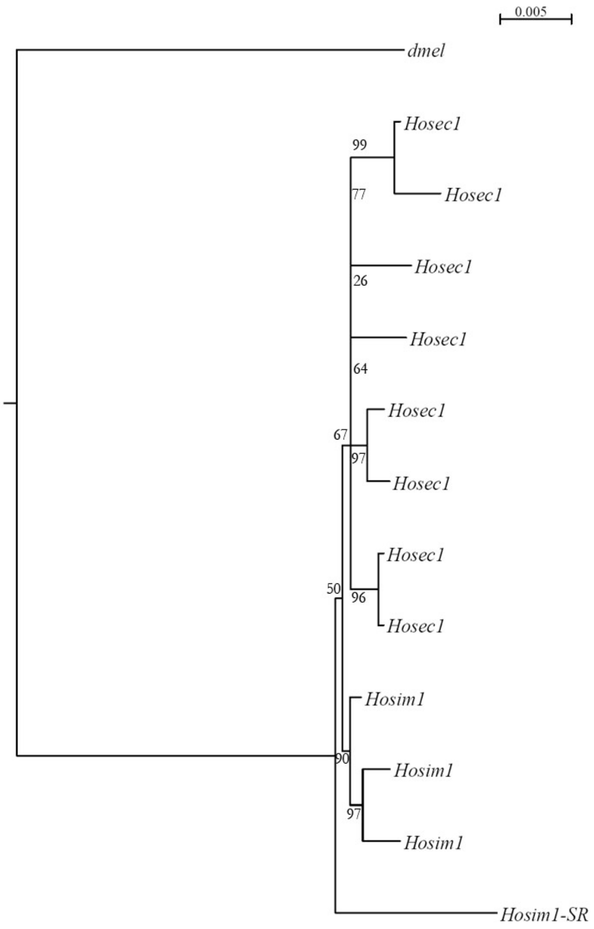

Figure S4 Maximum likelihood tree of *Hosim1* homologous sequences (HKY85 model) rooted with *D. melanogaster* sequence (*dmel*). *Hosec1 :* sequence found in *D. sechellia* (DE FREITAS ORTIZ and LORETO 2009). Numbers are the bootstrap values (100 repetitions).

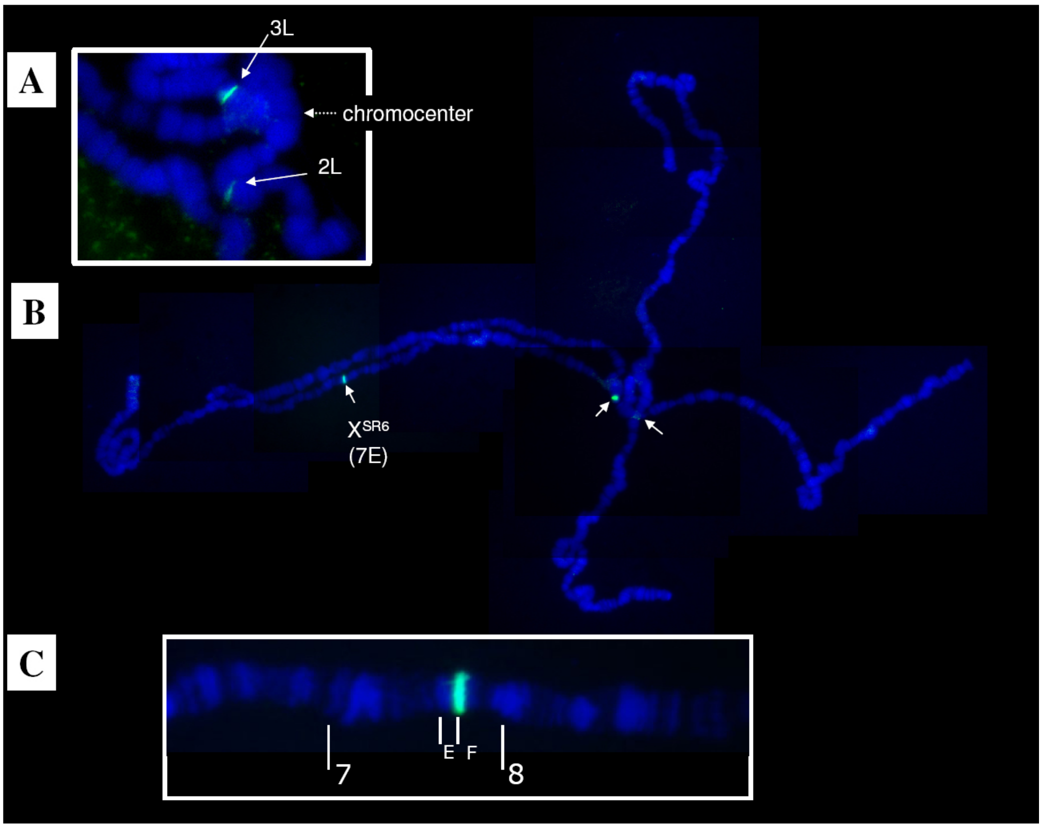

Figure S5 Visualization of *Hosim1* insertion sites by *in situ* hybridization on polytene chromosomes. (A) Two autosomal pericentromeric sites are visible in $(X^{ST8})_{ST8}$ males (3L and 2L arms). (B) In $(X^{SR6})_{ST8}$ males, an additional signal is visible on the X^{SR6} chromosome. It co-localizes with the supplementary band induced by the duplication (see Figure 2 in Montchamp-Moreau *et al.*, 2006). C. Focus on the X^{SR6} specific insertion site.

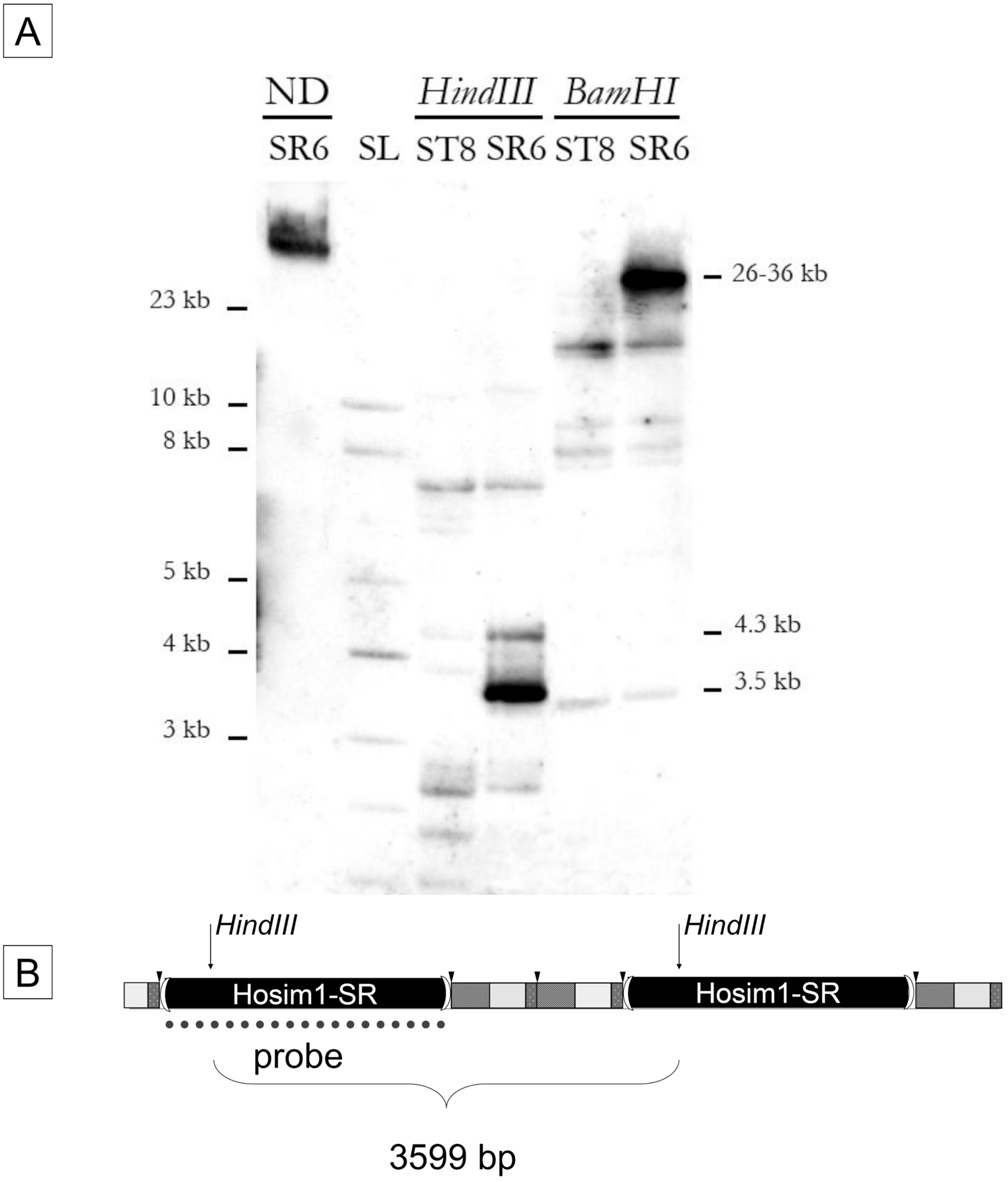

Figure S6 Control of the organization of the junction region. (A) Southern blot with DNA of $(X^{SR6})_{ST8}$ males digested with *BamHI* or *HindIII* and probed with *Hosim1-SR*. ND: Non Digested. ST8= $(X^{ST8})_{ST8}$ males, SR6 = $(X^{SR6})_{ST8}$ males. SL = Smart Ladder. (B) Position of *Hosim1* probe and *HindIII* sites in the basic module of the junction.

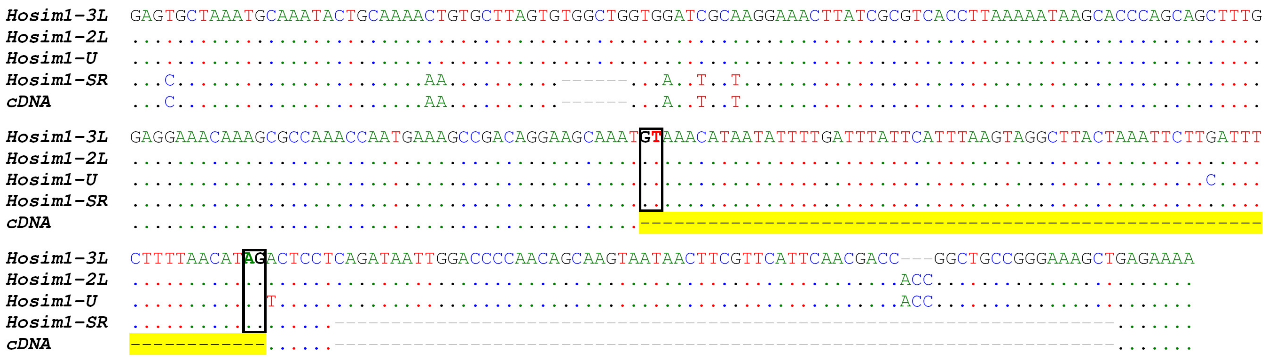

Figure S7 Identification of an intron in *Hosim1-SR*. The shorter cDNA fragment of Figure 8 have been sequenced and aligned with the sequences of *Hosim1* (de Freitas Ortiz and Loreto 2009) and *Hosim1-SR* . Comparing the sequences shows that the sequenced cDNA came from *Hosim1-SR* form. The part highlighted in yellow shows an intron delimited by the characteristic GT-AG nucleotides, which is close to the deletion of 79nt specific of *Hosim1-SR* (Figure 5).

Table S1 PCR primers used in the study.

Locus	Name	Primers	Used for	DNA size (bp)	cDNA size (bp)
GAPDH	GAPDH-q1	AAGGCTGGCATTTCGCTGAACG<br>AATTGCGCCCCTTGCGGATTATG	quantification	198	198
Hosim1	hAT1-F<br>hAT1-R	CGCCCCGGATACAAGATGCCCA<br>CTGCAACAAGTGCAGCAACACA	probe for FISH	850	850
	Hosim1-q1-F<br>Hosim1-q1-R<br>(c)	GGAGCTGGCATCTGCAGTTTGT<br>CTGCTCAACGAATGCCTCGCA	quantification	148	148
	Hosim1ST-q-F<br>Hosim1ST-q-R<br>(b)	TTCACCAAACTGAACGAACG<br>TAAGTGAACAACGAACGAACG	quantification	150	150
	hAT2-F<br>hAT2-R<br>(a)	ATGGCTTCAAAACGAAAGCA<br>CATTATCGGAAACCACAGCA	Length polymorphism Hosim1-ST Hosim1-SR	Hosim1-ST : 784<br>Hosim1-SR : 699	Hosim1-ST : 784 and 717<br>Hosim1-SR : 699 and 632
	Hosimbam-F<br>Hosimbam-R	GGATCCCCCGAGTGCGCAGGGAAGTT<br>GGATCCGGCACAGAAAATACGCAAGGAAGCA	probe for Southern blot		
RPL17	RPL17-q2-F<br>RPL17-q2-R	CCCTCCTTTTCGTTTTCGTT<br>GTGTTGTCGGCACAGTTCAT	quantification	345	161
RPII140	RPII140-q1-F<br>RPII140-q1-R	ATGGTGGCTTGCGTTTCGGTG<br>ATTGTTGCGCAGATTGGCGATGG	quantification	158	158
light	Light410-F<br>Light535-R	CCGATTCCAAAGCTCACATT<br>TTGACAAAACACTGCCTTCG	quantification	194	126
Trf2	DMTRF2-1<br>DMTRF2-2	GCCATCCATACACCACTGC<br>GAACGTTGCTTGCGGAAA	control of cDNA	526	462
IST	motif1-q2-F<br>motif1-q2-R	AGTTGCCTTTGAGTTTTCTG<br>ACTGATTTTGTTCAACTGACT	expression of *rISTs*	79	79
junction distal (a)	jct-F<br>jct-orgLongR2	GTCACTTGCAGGCAGACAAA<br>TGCGCACTCGGGGTCACGAT	control of duplication organization	757	-
junction proximal (c)	hosimSR1768-F<br>jctTRF2-R	ACATCAGTTCCTTGCGAACGCCT<br>AGAGAGTACGTGCCAGTGTTGA	control of duplication organization	1907	-
junction internal (b)	Jct-oranjbleu-F<br>hosimSR247-R	AGGGTACGGCAACGGTCACA<br>TCCTCCAAAGCTGCTGGGTGC	control of duplication organization	627	-
