# Supplementary material for: Sequence Analysis of the Segmental Duplication Responsible for Paris Sex-Ratio Drive in Drosophila simulans
Source: G3 (Bethesda). 2011 Oct 1;1(5):401–10. doi: 10.1534/g3.111.000315 (PMC3276153; doi:10.1534/g3.111.000315)
Supplement: Supporting Information [file supp_1.5.401_FigureS1.pdf]

A

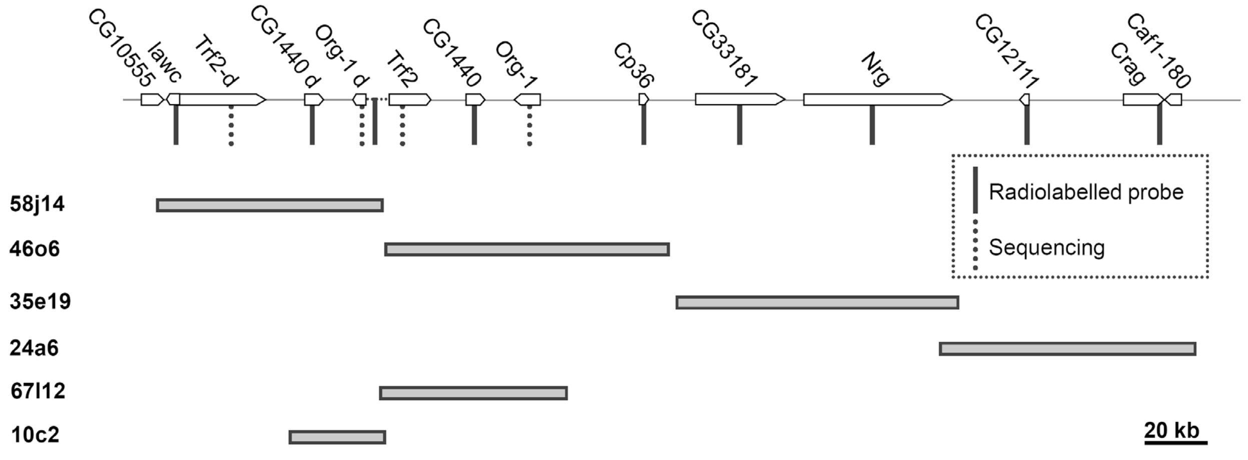

B

Locus	Name	Primers	Size bp
		Radiolabelled probes	
lawc	DMCG32711ex1-1<br>DMCG32711_1_5	TGTGTGAGTCGCGATAAACG<br>TCAGCTCTGGTAGCGAAACA	738
CG1440	DMCG1440ex35-1<br>DMCG1440ex35-2	GCGTTATGTTAAGCCGCATT<br>GGCGTTCATCACCAGATAGC	598
IST-Trf2	inter43_44-1<br>Inter43_44-2	AACCGGTTATGATGCGAAAG<br>CCGTATAGCTCAACCGATTTG	936
Cp36	CP36-F1<br>CP36-R1	CAGACAGCGAGCAGTAGACG<br>GTGGAGCCTCGATCTTCTTG	802
CG33181	DMCG33181-3<br>DMCG33181-4	TTCTACGGAATGCAAACGTG<br>TTGAGCGATACAATCGAACG	694
Nrg	NRGintron1F<br>NRGintron1R	GCAGTGCAGAAAGCAAAGGT<br>CCGGTTGTTTGGTGATTCTT	350
CG12111	DSC12111-F1<br>DSC12111-R1	TCTGCTGTTAATCCTGACGA<br>TACCACTTAATGTAGGTGAACTTGA	614
Crag	DMCRAG7<br>DMCRAG8	GTACAGATCGAACGGCAGGT<br>CATACTCGCGATGGAATGTG	805
		Sequencing probes	
Trf2	DMTRF2-1<br>DMTRF2-2	GCCATCCATACACCACTGC<br>GAACGTTGCTTGCGGAAA	530
Org-1	OrgF1<br>OrgR1	AGCCACTTTCGCACTTTCAT<br>GTGCCATCACATCCCGAAAG	1054

Figure S1 Selection of the clones used to sequence the *sex-ratio* region on X^{SR6}. (A): Schematic representation of the *sex-ratio* region on the X chromosome and position of the clones. For the sake of simplicity, we only represented the genes used to design probes for the selection of clones and two genes adjacent to the region under study. The open boxes represent the genes. The grey boxes represent the clones, with their names on the left. The vertical bars show the fragments used as probes to position the clones on the X chromosome (see materials and methods), the dotted vertical lines represent the fragments that were sequenced to discriminate between the proximal and distal copies of the duplication. (B): PCR primers to produce the fragments used as probes (ordered from left to right).
