# Supplementary material for: Sequence Analysis of the Segmental Duplication Responsible for Paris Sex-Ratio Drive in Drosophila simulans
Source: G3 (Bethesda). 2011 Oct 1;1(5):401–10. doi: 10.1534/g3.111.000315 (PMC3276153; doi:10.1534/g3.111.000315)
Supplement: Supporting Information [file supp_1.5.401_FigureS2.pdf]

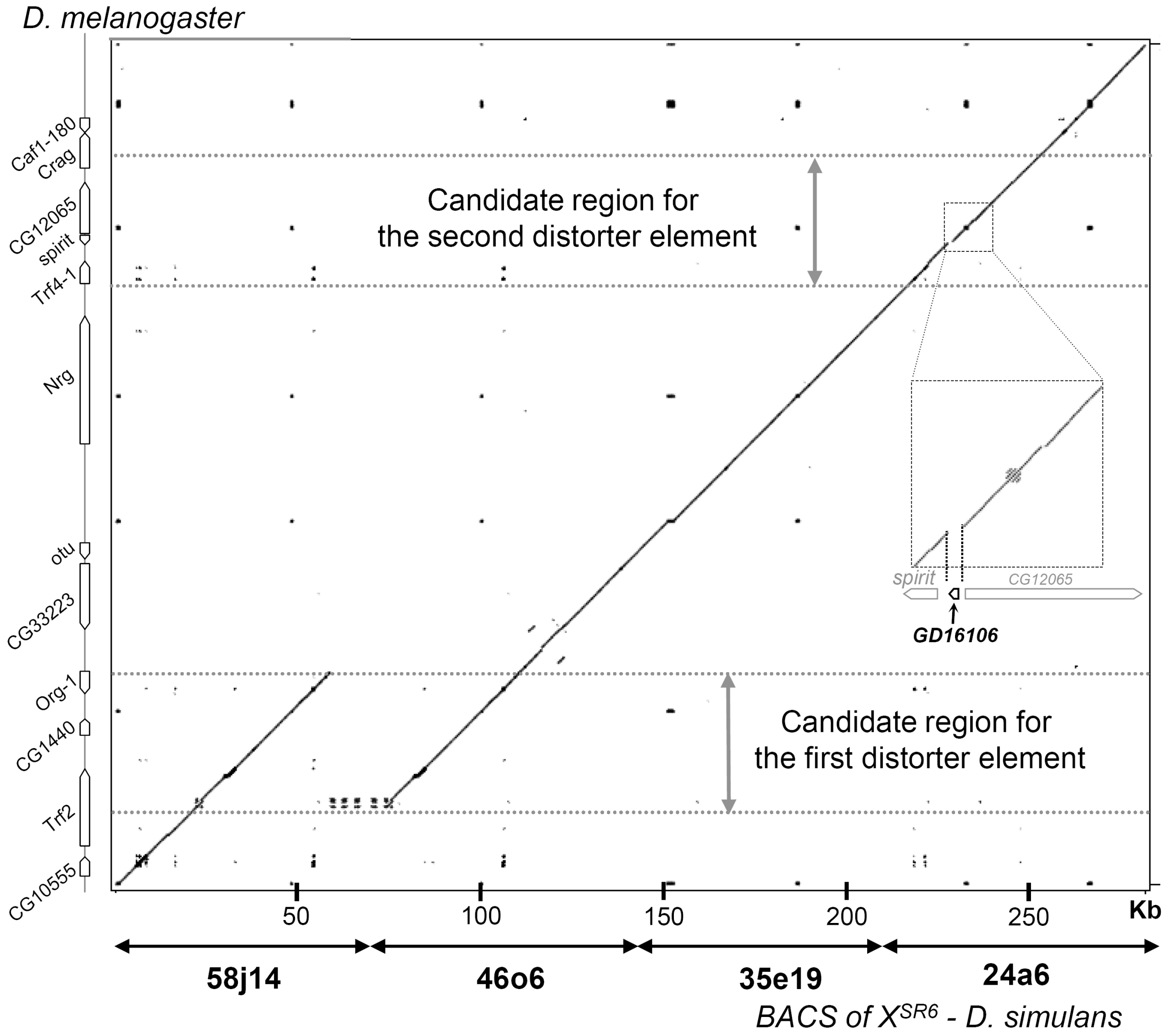

Figure S2 Dot plot comparison of the *sex-ratio* region on the X^{SR6} chromosome of *D. simulans* (below) with the homologous region in *D. melanogaster* (left). Boxes along the vertical axis represent the genes annotated in *D. melanogaster* genome. The limits of the candidate region for the second element involved in drive are those determined in Montchamp-Moreau *et al* (2006).
