# Supplementary material for: Sequence Analysis of the Segmental Duplication Responsible for Paris Sex-Ratio Drive in Drosophila simulans
Source: G3 (Bethesda). 2011 Oct 1;1(5):401–10. doi: 10.1534/g3.111.000315 (PMC3276153; doi:10.1534/g3.111.000315)
Supplement: Supporting Information [file supp_1.5.401_FigureS4.pdf]

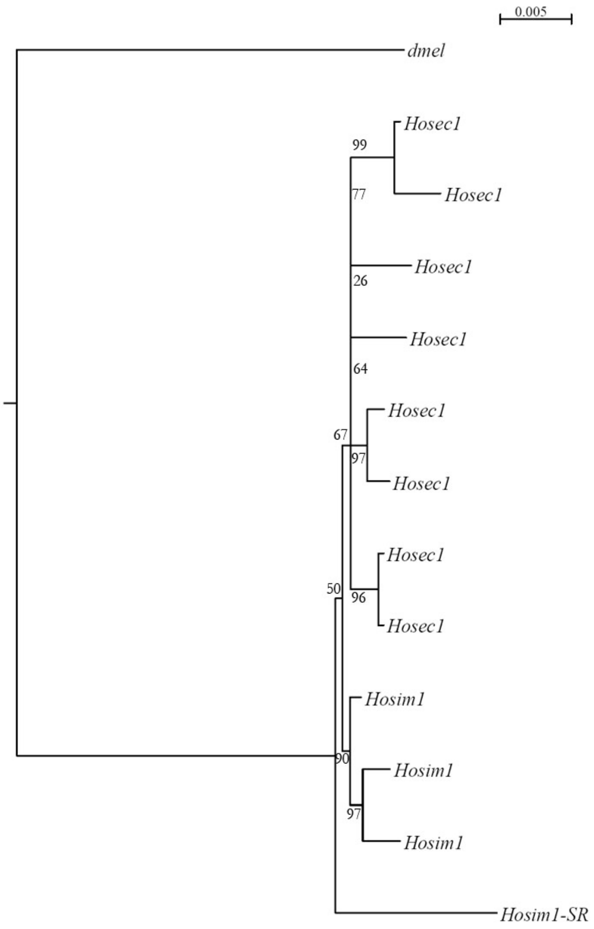

Figure S4 Maximum likelihood tree of *Hosim1* homologous sequences (HKY85 model) rooted with *D. melanogaster* sequence (*dmel*). *Hosec1 :* sequence found in *D. sechellia* (DE FREITAS ORTIZ and LORETO 2009). Numbers are the bootstrap values (100 repetitions).
