# Supplementary material for: Sequence Analysis of the Segmental Duplication Responsible for Paris Sex-Ratio Drive in Drosophila simulans
Source: G3 (Bethesda). 2011 Oct 1;1(5):401–10. doi: 10.1534/g3.111.000315 (PMC3276153; doi:10.1534/g3.111.000315)
Supplement: Supporting Information [file supp_1.5.401_FigureS5.pdf]

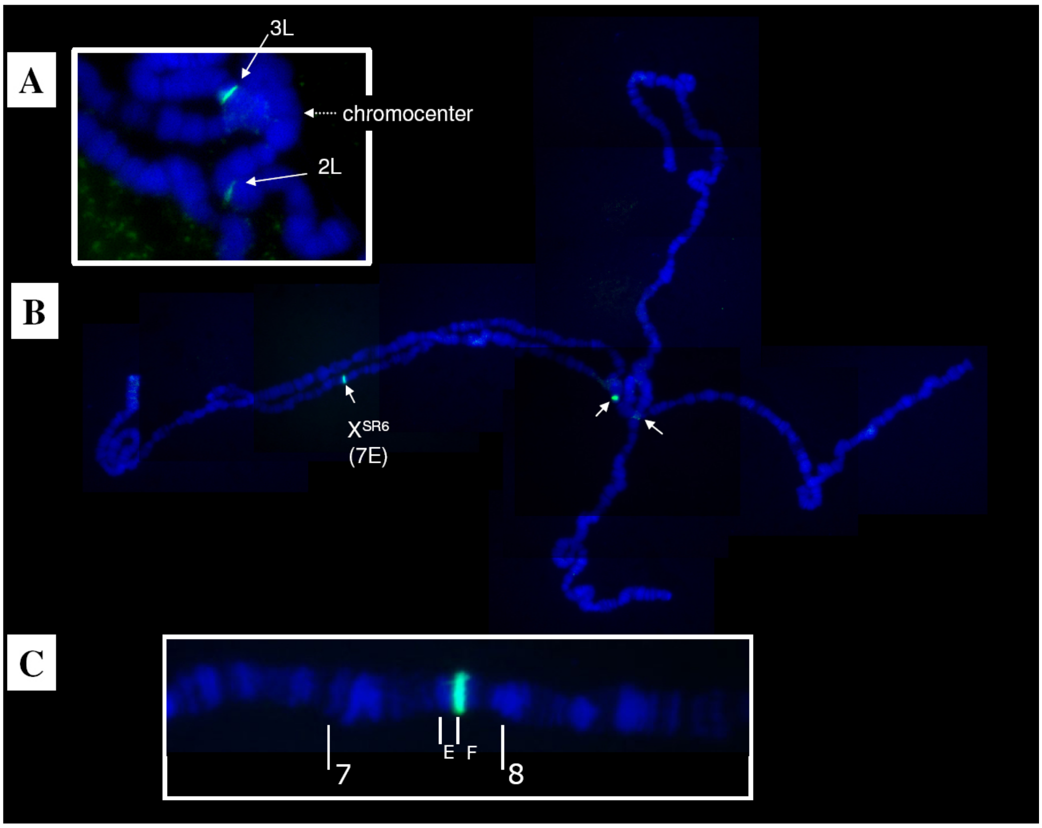

Figure S5 Visualization of *Hosim1* insertion sites by *in situ* hybridization on polytene chromosomes. (A) Two autosomal pericentromeric sites are visible in $(X^{ST8})_{ST8}$ males (3L and 2L arms). (B) In $(X^{SR6})_{ST8}$ males, an additional signal is visible on the X^{SR6} chromosome. It co-localizes with the supplementary band induced by the duplication (see Figure 2 in Montchamp-Moreau *et al.*, 2006). C. Focus on the X^{SR6} specific insertion site.
