# Supplementary material for: Sequence Analysis of the Segmental Duplication Responsible for Paris Sex-Ratio Drive in Drosophila simulans
Source: G3 (Bethesda). 2011 Oct 1;1(5):401–10. doi: 10.1534/g3.111.000315 (PMC3276153; doi:10.1534/g3.111.000315)
Supplement: Supporting Information [file supp_1.5.401_FigureS6.pdf]

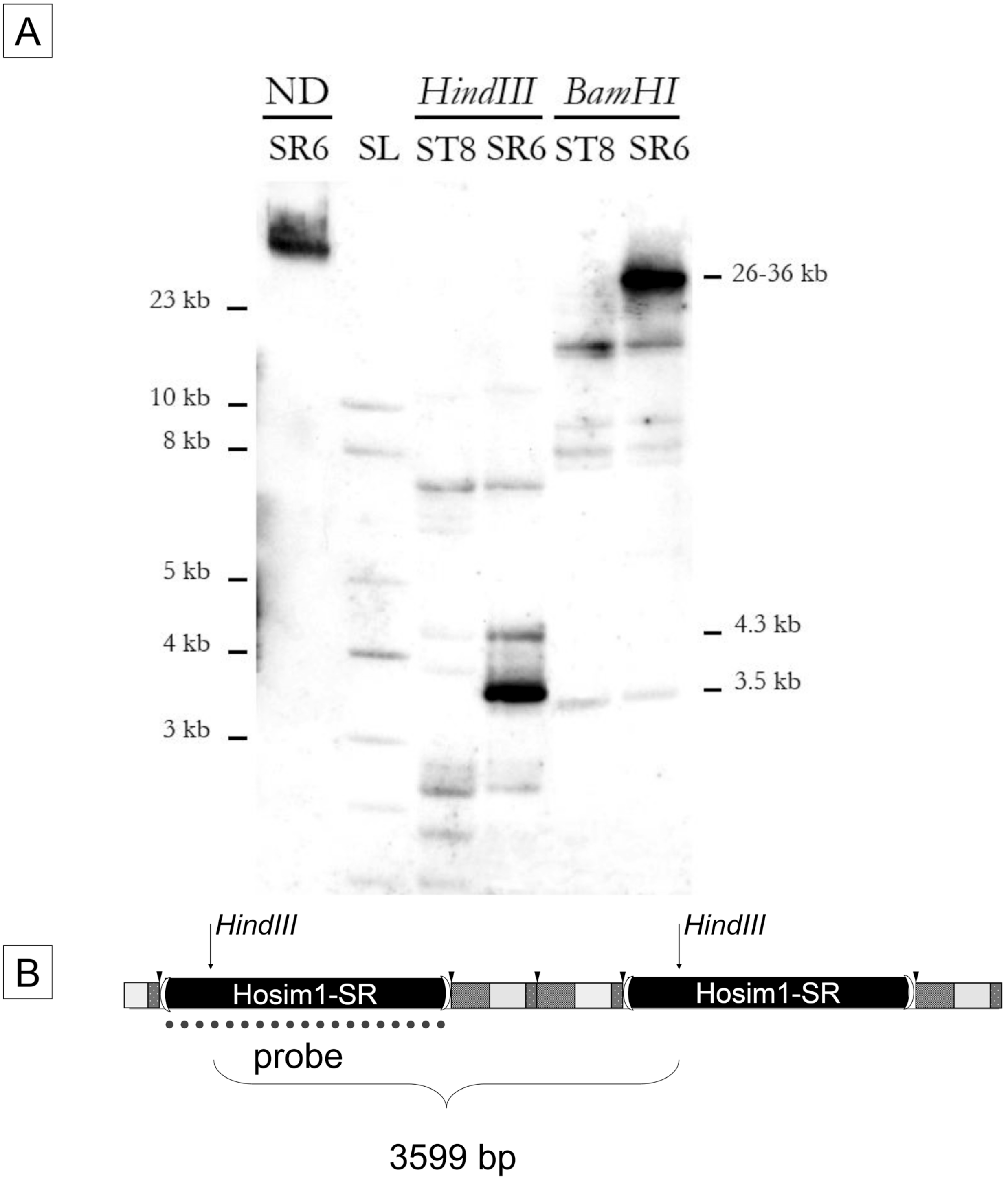

Figure S6 Control of the organization of the junction region. (A) Southern blot with DNA of $(X^{SR6})_{ST8}$ males digested with *BamHI* or *HindIII* and probed with *Hosim1-SR*. ND: Non Digested. ST8= $(X^{ST8})_{ST8}$ males, SR6 = $(X^{SR6})_{ST8}$ males. SL = Smart Ladder. (B) Position of *Hosim1* probe and *HindIII* sites in the basic module of the junction.
