# Supplementary material for: Sequence Analysis of the Segmental Duplication Responsible for Paris Sex-Ratio Drive in Drosophila simulans
Source: G3 (Bethesda). 2011 Oct 1;1(5):401–10. doi: 10.1534/g3.111.000315 (PMC3276153; doi:10.1534/g3.111.000315)
Supplement: Supporting Information [file supp_1.5.401_FigureS7.pdf]

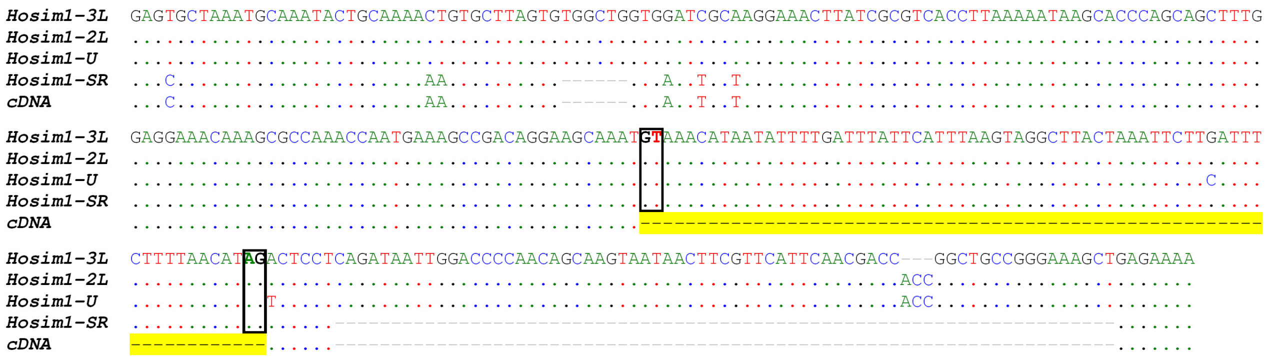

Figure S7 Identification of an intron in *Hosim1-SR*. The shorter cDNA fragment of Figure 8 have been sequenced and aligned with the sequences of *Hosim1* (DE FREITAS ORTIZ and LORETO 2009) and *Hosim1-SR* . Comparing the sequences shows that the sequenced cDNA came from *Hosim1-SR* form. The part highlighted in yellow shows an intron delimited by the characteristic GT-AG nucleotides, which is close to the deletion of 79nt specific of *Hosim1-SR* (Figure 5).
