# Supplementary material for: Sequence Analysis of the Segmental Duplication Responsible for Paris Sex-Ratio Drive in Drosophila simulans
Source: G3 (Bethesda). 2011 Oct 1;1(5):401–10. doi: 10.1534/g3.111.000315 (PMC3276153; doi:10.1534/g3.111.000315)
Supplement: Supporting Information [file supp_1.5.401_TableS1.pdf]

Table S1 PCR primers used in the study.

Locus	Name	Primers	Used for	DNA size (bp)	cDNA size (bp)
GAPDH	GAPDH-q1	AAGGCTGGCATTTCGCTGAACG<br>AATTGCGCCCCTTGCGGATTATG	quantification	198	198
Hosim1	hAT1-F<br>hAT1-R	CGCCCCGGATACAAGATGCCCA<br>CTGCAACAAGTGCAGCAACACA	probe for FISH	850	850
	Hosim1-q1-F<br>Hosim1-q1-R<br>(c)	GGAGCTGGCATCTGCAGTTTGT<br>CTGCTCAACGAATGCCTCGCA	quantification	148	148
	Hosim1ST-q-F<br>Hosim1ST-q-R<br>(b)	TTCACCAAACTGAACGAACG<br>TAAGTGAACAACGAACGAACG	quantification	150	150
	hAT2-F<br>hAT2-R<br>(a)	ATGGCTTCAAAACGAAAGCA<br>CATTATCGGAAACCACAGCA	Length polymorphism Hosim1-ST Hosim1-SR	Hosim1-ST : 784<br>Hosim1-SR : 699	Hosim1-ST : 784 and 717<br>Hosim1-SR : 699 and 632
	Hosimbam-F<br>Hosimbam-R	GGATCCCCCGAGTGCGCAGGGAAGTT<br>GGATCCGGCACAGAAAATACGCAAGGAAGCA	probe for Southern blot		
RPL17	RPL17-q2-F<br>RPL17-q2-R	CCCTCCTTTTCGTTTTCGTT<br>GTGTTGTCGGCACAGTTCAT	quantification	345	161
RPII140	RPII140-q1-F<br>RPII140-q1-R	ATGGTGGCTTGCGTTTCGGTG<br>ATTGTTGCGCAGATTGGCGATGG	quantification	158	158
light	Light410-F<br>Light535-R	CCGATTCCAAAGCTCACATT<br>TTGACAAAACACTGCCTTCG	quantification	194	126
Trf2	DMTRF2-1<br>DMTRF2-2	GCCATCCATACACCACTGC<br>GAACGTTGCTTGCGGAAA	control of cDNA	526	462
IST	motif1-q2-F<br>motif1-q2-R	AGTTGCCTTTGAGTTTTCTG<br>ACTGATTTTGTTCAACTGACT	expression of *rISTs*	79	79
junction distal (a)	jct-F<br>jct-orgLongR2	GTCACTTGCAGGCAGACAAA<br>TGCGCACTCGGGGTCACGAT	control of duplication organization	757	-
junction proximal (c)	hosimSR1768-F<br>jctTRF2-R	ACATCAGTTCCTTGCGAACGCCT<br>AGAGAGTACGTGCCAGTGTTGA	control of duplication organization	1907	-
junction internal (b)	Jct-oranjbleu-F<br>hosimSR247-R	AGGGTACGGCAACGGTCACA<br>TCCTCCAAAGCTGCTGGGTGC	control of duplication organization	627	-
