# Supplementary material for: Sequence Analysis of the Segmental Duplication Responsible for Paris Sex-Ratio Drive in Drosophila simulans
Source: G3 (Bethesda). 2011 Oct 1;1(5):401–10. doi: 10.1534/g3.111.000315 (PMC3276153; doi:10.1534/g3.111.000315)
Supplement: Supporting Information [file supp_1.5.401_FigureS3.pdf]

DDSA traces interpretation			
**Site**	**Position**	**DDSA type of trace**	**Deduction of the neo-synthesized copy**
1	5838	Tandem repeat	Proximal copy (46o6)
2	5949	Micro-homology traces	Proximal copy (46o6)
3	6198	Tandem repeat	Distal copy (58j14)
4	6992	Micro-homology traces	Proximal copy (46o6)
5	8787	Micro-homology traces	Proximal copy (46o6)

**Legends:**
tandem repeat
micro-homology traces

Site 1: Position: 5838 bp

```
XSR6d-58j14   CCGGCATTTATCAAAACGGCAATGTCG---------GTGGAATCTTTAACTAGAAACAAC
XSR6p-46o6    CCGGCATTTATCAAAACGGCAATGTCGTGGAATCGTGTGGAATCTTTAACTAGAAACAAC
              ***************************         ************************

Dsim          CCGGCATTTATCAAAACGGCAATGTCG---------GTGGAATCTTTAACTAGAAACAAC
Dsec          CCGGCATTTATCAAAACGGCAATGTCG---------GTGGAATCTTTAACTAGAAACAAC
Dmel          GAGGCATTTATCAAATCGGCAATGTCG---------GGGGAATCTTCAACTAGAAACAAC
```

Site 2: Position: 5949 bp

```
XSR6d-58j14   ATAGATCTCTTTGGCATTAAAT------GAGTGAAGCTCCTAAATGTACATATGCAATAT
XSR6p-46o6    ATAGATCTCTTTGGC----------------------TCCTAAATGTACATATGCAATAT
              ***************                      ***********************

Dsim          ATAGATCTCTTTGGCATTAAAT------GAGTGAAGCTCCTAAATGTACATATGCAATAT
Dsec          ATAGATCTCTTTGGCATTAAATTAAAATGAGTGAAGCTCTTAAATGTACATATGCAATAT
Dmel          ATAGATCTTTTTGGCATTAAAT------GAGTGAAGCTCCTAAACATACATATGCAATAT
Dyak          ATAGACCTTTTTGGTATTAAAT------GAGAGAAGCTCCTAAATGTACATTTGAAATAT
```

Site 3: Position: 6198 bp

```
XSR6d-58j14   TCGATGTTGGTGCATAACTGTTGTTGTTGCATAACTGTTGTTGTTGCTGTTGCTCGATTT
XSR6p-46o6    TCGATGTTGGTGCATAACTGTTGTTGTTGC-----------------TGTTGCTCGATTT
              ******************************                 *************

Dsec          TCGATGTTGGTGCATAACTGTTGTTGTTGC-----------------TGTTGCTCGATTT
Dsim          TCGATGTTGGTGCATAACTGTTGTTGTTGC-----------------TGTTGCTCGATTT
Dmel          TCGATGTTGGTGCATAACTGTTGTTGTTG--------------------TTGCTTGATTT
```

Site 4: Position: 6992 bp

```
XSR6d-58j14   AATTTGCGTGTTCTTATTTTCCCAAATGTCAACTTTTCCAATTATTTATTTACTTTTTAT
XSR6p-46o6    AATTTACGTGTTCTTATTTTCCCAA----------------TTATTTATTTACTTTTTAT
              ***** *******************                *******************

Dsim-a        AATTTGCGTGTTCTTATTTTCCCAAATTTCAACTTTTCCAATTATTTATTTACTTTTTAT
Dsim-b        AATTTGCGTGTTCTTATTTTCCCAAATGTCAACTTTTCTAATTATTTATTTACTTTTTAT
Dsec          AATTTGCATGTTCTTATTTTCCCAAATGTCAACTTTTCCGATTATTTATTTACTTTTGAT
Dmel          AATTTGCATGTTTTCATTTTCCCAAATGTCAACTTTACGA-----TTATTAACTTTTATT
```

Site 5: Position: 8787 bp

```
XSR6d-58j14   GAGTACCAGGATTCAAATCACGAGTACCAGGACTTGAATCACGAGTATCAGGACTCAACA
XSR6p-46o6    GAGTACCAGGATTCAA---------------------ATCACGAGTATCAGGACTCAACA
              ****************                     ***********************

Dsim          GAGTACCAGGATTCAAATCACGAGTACCAGGACTTGAATCACGAGTATCAGGACTCAACA
```

Figure S3 Signatures of the DDSA model. Several traces of the DDSA model have been detected when aligning the duplicated fragments according to Fiston-Lavier *et al* (2007): XSR6d-58j14 = distal copy on X^{SR6}, XSR6p-58j14 = proximal copy on X^{SR6}. The stars show the position identical between the copies. Homologous sequences in other species were found in flybase. Dsim: *D. simulans*, Dsec: *D. sechellia*, Dmel: *D. melanogaster*, and *Dyak*: *D. yakuba.* The positions of sites correspond to those in abscissa of Figure 2.
